# Well-Being Outcomes of Health Care Workers After a 5-Hour Continuing Education Intervention: The WELL-B Randomized Clinical Trial

**DOI:** 10.1001/jamanetworkopen.2024.34362

**Published:** 2024-09-19

**Authors:** J. Bryan Sexton, Kathryn C. Adair

**Affiliations:** 1 Department of Psychiatry, Duke University School of Medicine, Duke University Health System, Durham, North Carolina; 2 Duke Center for the Advancement of Well-being Science, Duke University Health System, Durham, North Carolina

## Abstract

**Importance:**

Compromised well-being in health care workers (HCWs) is detrimental to the workforce, organizations, and patients.

**Objective:**

To test the effectiveness of Well-Being Essentials for Learning Life-Balance (WELL-B), a web-based continuing education program to deliver brief, evidence-based, reflective, psychological interventions to improve 4 dimensions of HCW well-being (ie, emotional exhaustion, emotional thriving, emotional recovery, and work-life integration).

**Design, Setting, and Participants:**

A randomized clinical trial (RCT) of US inpatient and outpatient HCWs randomized 1:1 was conducted from January 3 through May 31, 2023, using a web-based intervention. Cohort 1 received 5 hours of WELL-B over 8 days; cohort 2 acted as the control group and received WELL-B after the end of the RCT. Eligibility criteria were US HCWs aged 18 years or older, including clinical (physician, nurse, and respiratory therapist) and nonclinical (administrative, information technology, and finance) roles.

**Interventions:**

Continuing education sessions exposed participants to positive psychology interventions (gratitude letter, work-life integration, self-compassion and cultivating awe).

**Main Outcomes and Measures:**

The primary outcome was emotional exhaustion on day 8; secondary outcomes included emotional thriving, emotional recovery, and work-life integration. All outcomes were measured using psychometrically valid scales previously reported in well-being RCTs and were assessed on days 1 and 8 (primary end point). Differences in outcome measures between the WELL-B intervention group and controls were assessed using *t* tests. Baseline-adjusted multiple linear regression models were evaluated to examine the association between the WELL-B intervention and the outcome measures after adjusting for additional covariates (sex, race and ethnicity, age, HCW role, and discipline). Intention-to-treat analysis was performed.

**Results:**

The cohorts were similar at baseline, mostly female (528 [89%]) and nurses (177 [30%]). A total of 643 respondents participated in the study. In cohort 1, 331 participants initiated WELL-B, and 262 (71%) completed the day 8 follow-up; in cohort 2, 312 participants initiated WELL-B and 291 (77%) completed the day 8 follow-up. Compared with the control cohort, WELL-B significantly improved emotional exhaustion (estimate: −9.0; 95% CI, −13.1 to −4.9; *P* < .001), emotional thriving (estimate: 6.6; 95% CI, 3.2-10.0; *P* < .001), emotional recovery (estimate: 5.5; 95% CI, 2.0-9.0; *P* = .002), and work-life integration (estimate: −5.0; 95% CI, −8.2 to −1.9; *P* = .002). After adjusting for baseline outcome measures, sex, race and ethnicity, age, HCW role, and discipline, the linear regression model showed WELL-B improved day 8 emotional exhaustion (estimate: −9.6; 95% CI, −12.5 to −6.6; *P* < .001) compared with the control group. Favorable impressions of WELL-B were reported by more than 90% of the participants.

**Conclusions and Relevance:**

In this RCT, brief well-being activities delivered during continuing education sessions improved short-term HCW emotional exhaustion, emotional thriving, emotional recovery, and work-life integration, with and without adjusting for covariates. Health care worker impressions of WELL-B were positive. These findings suggest that WELL-B is a beneficial intervention.

**Trial Registration:**

ClinicalTrials.gov Identifier: NCT05636072

## Key Points

**Question:**

Does a 5-hour web-based continuing education intervention (Well-Being Essentials for Learning Life-Balance [WELL-B]) improve health care worker well-being?

**Findings:**

In this randomized clinical trial including 643 health care workers, improvements in emotional exhaustion, emotional thriving, emotional recovery, and work-life integration were noted with WELL-B. Favorable impressions of WELL-B were reported by more than 90% of the participants.

**Meaning:**

The findings of this trial suggest that WELL-B is a brief, evidence-based continuing education intervention that may improve health care worker-well-being.

## Teaser

This randomized clinical trial assesses the use of a brief continuing education program designed to improve well-being in health care workers.

## Introduction

Health care worker (HCW) well-being was a key factor in care quality before the COVID-19 global health crisis.^1,2,3,4^ The aftermath of the COVID-19 pandemic has placed health care leaders into a position of managing shortfalls in budgets, staffing, and a substantial deterioration of HCW well-being.^5,6,7^ Compromised well-being in HCWs is expensive,^8^ and their struggles with work-life integration, challenges with the electronic health record, and difficult work cultures remain largely unaddressed.^9,10,11^

Many HCWs have become jaded by well-intentioned but poorly timed efforts to introduce meditation, yoga, diet, and exercise programs to a weary workforce. Despite the extensive evidence supporting a meditation practice,^12,13^ for example, the initiation energy required can feel overwhelming. Moreover, it is impractical and potentially offensive to tell HCWs coming off 18 hours of service that they need to clear their mind and focus on their breathing before starting work again in 2 hours. Ideally, well-being infrastructure and resources would be ubiquitous, include only evidence-based options, and be easily accessible and widely used. Until this is realized, interim solutions should make resources more accessible by incorporating them into daily activities, such as meetings, huddles, and continuing education offerings. One consequence of diminishing HCW well-being has been the rise of “bite-sized” interventions^14,15,16,17,18^ that are simple, brief, accessible, and feasible for busy HCWs. The science and study of well-being and human flourishing is known as “positive psychology” and has led to the development of positive psychology interventions that have been adapted for use by HCWs.^14,15,16,17,18^

Increasingly, evidence from animal models and humans suggests that stresses such as trauma surgery, disease, pregnancy, and even severe COVID-19 can cause increases in biological age compared with chronological age,^19,20^ all of which are reversible if the individual is provided sufficient time to recover. Time to recover is a difficult intervention to provide an underfunded, overextended, tired, and distracted workforce. Nevertheless, many HCWs are required to accrue routine continuing education credits, so we adapted a series of evidence-based bite-sized well-being interventions^17,18^ that could be administered during a continuing education program. The Well-Being Essentials for Learning Life-Balance (WELL-B) program provides 5 hours of credit to HCWs, and 10 minutes of each hour is allotted to engaging in a well-being intervention.

WELL-B uses updated versions of evidence-based interventions drawn from positive psychology that have been used to improve well-being and reduce depression symptoms, delivered via a mobile platform.^14,15,16,17,18^ The criteria used to select bite-sized interventions for WELL-B were that the activity be brief and simple, recovery is achieved quickly, and the benefits endure (6 and 12 months postintervention). Moreover, intervention uptake by busy HCWs has been increased due to supporting evidence, ease of use, engaging content, and personal relevance.^17,18^

The objective of this study was to assess the effect of positive psychology interventions embedded in continuing education sessions on 4 dimensions of well-being (emotional exhaustion, work-life integration, emotional thriving, and emotional recovery). This study used an individual-level randomized design in which participants were randomized into 1 of 2 cohorts—intervention or control. The efficacy of WELL-B was tested using the randomized trial day 8 end point.

The first aim was to test the hypothesis that the WELL-B intervention will improve HCW well-being, compared with the control group. The second aim was to examine the magnitude of the effect of the WELL-B intervention after adjusting for baseline outcome measures, sex, race and ethnicity, age, HCW role, and discipline. Race and ethnicity were included because these demographic variables have been linked to differences in HCW well-being.

## Methods

### Design

WELL-B was an investigator-initiated RCT of HCWs randomized (1:1) to 2 cohorts on enrollment: treatment (cohort 1) or control (cohort 2) and provided video conferencing links unique to their cohort. Participants enrolled in advance of the start date and were not aware of their cohort status because the same start date was given to both cohorts. On the start date, separate video conferencing (Zoom Video Communications Inc) sessions were used for each cohort to receive a study overview and complete baseline well-being measures, including information about their well-being, sleep quantity and quality, an assessment of their well-being, and personalized feedback about their well-being. Cohorts were primarily assessed at 2 points: baseline and after completing the 8-day intervention. Cohort 1 was assessed on day 1 (baseline) and day 8 (post-RCT). At the end of the RCT, cohort 2 received the intervention, so cohort 2 was assessed on day 1 (baseline), day 8 (post-RCT), and day 15 (postintervention). Participants were blinded to their cohort status and investigators were not. WELL-B was described as a brief continuing education intervention, without any long-term commitment. The wherewithal necessary for HCWs to initiate and complete well-being activities is increasingly challenging^7,21^; therefore, we used the postintervention results (8 days of intervention) as the primary end point to facilitate recruitment and minimize attrition (recruitment began after the Omicron wave of COVID-19). An online survey platform (Qualtrics, Silver Lake and CPP Investments) was used for enrollment, data collection, randomization of individual enrollees to study cohort, and delivery of video conferencing links to the WELL-B continuing education sessions. Duke University Health System Institutional Review Board approved the trial protocol and statistical analysis plan (Supplement 1) for this RCT. We followed the Consolidated Standards of Reporting Trials (CONSORT) reporting guideline for randomized trials. Participants provided a web-based informed consent, and there were was no financial compensation.

### Participants

Inpatient and outpatient HCWs within the US enrolled January 3 through May 31, 2023, using a link on the Duke Center for the Advancement of Well-Being Science (CAWS) website,^22^ or were provided the link during grand rounds, continuing education talks, and monthly well-being sessions offered by CAWS. Generally, people who seek content on the website and/or attend these continuing education activities have a background or interest in patient safety, safety culture, quality improvement, and/or well-being. There was also a brief explanation of the WELL-B intervention during enrollment that provided an overview of the prevalence and severity of well-being issues in health care, as well as the length and nature of WELL-B, which was termed a method for pausing and reflecting on what is going well.

### Intervention

WELL-B comprises 5 guided well-being modules based on adult learning principles, combining educational material with practice-based learning.^17^ Individual modules have been favorably evaluated as brief, feasible, and practical.^16,18,20^ WELL-B was delivered as 5 continuing education hours, each of which contained 10 minutes of interactive content with participants as part of the research, assessment, feedback and tool (RAFT) format. Generally, the first 30 minutes presented current evidence on HCW well-being and the topic of the session (eg, work-life integration), followed by a 10-minute portion during which the participant completed a well-being assessment (eg, frequency of work-life behaviors in the past week), received feedback and benchmarking about the assessment, and completed the brief intervention (tool) for that session. The final 10 to 15 minutes reflected additional evidence on the topic of well-being explored during that session, followed by 5 to 10 minutes of moderated questions and answers. Participants used a quick response code presented during the session to access the assessment, feedback, and tool portions of RAFT. Aside from enrolling in the study, there was no prework or postwork required of participants. Based on extensive feedback from Duke Monthly Well-Being Webinar Series^23^ participants, optimal participation would be achieved at 12:00 to 1:00 pm, local time, Monday through Thursday; therefore, WELL-B ran for 8 days, Monday through Thursday and the following Monday. The order of the modules was as follows:

Session 1: Gratitude as Easy Well-Being: New Science on an Old Practice,
Session 2: Work-Life Integration: Measuring & Understanding HCW Well-Being,
Session 3: The Voice in Your Head Isn’t Always Kind: Evidence-Based Self-Compassion,
Session 4: Science of Wow: Cultivating Awe and Wonder as a Well-Being Strategy, and
Session 5: Group-Level Well-Being, Follow-Up, Sharable Resources, and Extended question and answer.

Session 1 (gratitude) provided a structured opportunity to select a person and express gratitude toward that individual through a guided letter-writing exercise.^24,25^ Session 2 (work-life integration) asked participants to reflect on their own behaviors, prompted them with a reminder during each of the 5 sessions, and asked them to prioritize simple actions in favor of their well-being.^9,10,16,24,26^ Session 3 (self-compassion) provided a guided opportunity to pause and reflect on how we treat ourselves, using deliberate language as if talking to a friend.^26^ Session 4 (cultivating awe) provided an opportunity to learn about and experience the benefits of awe and wonder through a series of visually and conceptually stunning images followed by an exercise to reflect on one of their own experiences of awe.^17,18,27^ Session 5 (group-level well-being) demonstrated the role of well-being informed leadership, psychological safety,^28^ leader well-being check-ins,^29,30^ and positive rounding.^31^

### Measures

The multidimensional nature of well-being makes it difficult to summarize in 1 domain. Randomized clinical trial results have demonstrated the utility of 4 domains that were used for the present study: emotional exhaustion, emotional thriving, emotional recovery, and work-life integration. Given the contemporary relevance to HCWs, responsiveness to interventions, and psychometric validity, we chose the primary outcome of emotional exhaustion. Emotional exhaustion was assessed by a widely used^7,9,10,14,16,32,33^ 5-item derivative scale of the emotional exhaustion scale of the Maslach Burnout Inventory,^34^ reported to have excellent psychometric properties,^14,15,16,32,33,35,36^ external validity,^9,10,32^ and responsiveness to interventions.^11,14,15,16,35^ Compared with the 9-item emotional exhaustion scale, the 5-item derivative scale was observed to have equivalent or superior convergent validity and generates superior model fit.^37^ These metrics are associated with fear of making clinical errors an HCW mental health.^38^ Details of each well-being domain are described by Sexton et al.^18^ For comparability across the 4 well-being domains, we rescaled outcome measures to 100-point scales, consistent with previous studies.^7,14,15,16,17,18,26^ For example, emotional exhaustion items use a 5-point Likert scale ranging from disagree strongly (1) to agree strongly (5). Using the mean of the emotional exhaustion items for each participant (ranging from 1 to 5), we subtracted 1 and multiplied it by 25, rescaling the range from 0 to 100 (with higher scores indicating more severe emotional exhaustion). Consistent with earlier research, a score less than 50 indicates no emotional exhaustion (on average, disagreeing slightly or strongly with all emotional exhaustion items), 50 to 74 indicates mild emotional exhaustion (on average, being neutral or agreeing slightly), 75 to 95 indicates moderate emotional exhaustion (on average, agreeing slightly or strongly), and greater than 95 indicates severe emotional exhaustion (agreeing strongly with all items). Scores of 50 or greater indicate concerning levels of emotional exhaustion, because the respondent is not disagreeing with the exhaustion items.

### Randomization

Participants enrolled using the survey platform (Qualtrics), which consecutively randomized participants into 2 cohorts. Participants received details of the intervention and their start date in an email for their records.

### Statistical Analysis

Characteristics of the study population before the intervention were summarized using mean (SD) or frequency with percentage, where appropriate. The Cronbach α was used as a rough gauge of well-being scale psychometric reliability (ranging between 0 and 1, with values of 0.7 and higher indicating acceptable reliability). To assess the efficacy of WELL-B (aim 1), equal variance *t* tests were conducted to assess differences at the end of the RCT (day 8) measures for cohorts 1 and 2 for each of the outcomes of interest. Additional paired *t* tests were assessed to compare outcome measures for cohort 2 after completing the WELL-B intervention (day 8 vs day 15). Baseline-adjusted multiple linear regression models were examined (aim 2) to assess the association between the WELL-B intervention and day 8 measures after adjusting for key covariates (sex, race and ethnicity, age, HCW role, and discipline). Robust (sandwich) estimates of variance were used to calculate test statistics. Baseline-adjusted multiple linear regression models were also evaluated using percent-concerning thresholds as an additional way to assess the magnitude of the intervention effect (aim 2). Percent-concerning thresholds are commonly used in safety culture and well-being research when looking across a set of metrics that have different valances. For example, emotional exhaustion and work-life integration are negatively valanced, whereas emotional thriving and emotional recovery are positively valanced, so presenting a lower percent-concerning value was a positive result across all 4 well-being domains for ease of interpretation.^9,10,32,39^

All data analysis was performed in SAS, version 9.4 (SAS Institute Inc). *P* < .05 was considered statistically significant. The number of participants in cohorts 1 and 2 is able to detect an effect size of 0.21 with 80% power for aim 1. We considered emotional exhaustion improvement of at least 10% from baseline to be meaningful based on previous studies.^15,16,17,40^ This translated to a decrease in emotional exhaustion from 50 of 100 to 45 of 100, a 5-point decrease, or an effect size of 0.25, assuming an SD of 20. For aim 2, at least 405 participants were needed to detect a small effect size of 0.02 for the WELL-B intervention in the baseline-adjusted multiple linear regression models. Analyses were conducted with the intention-to-treat approach.

## Results

**Figure 1.**
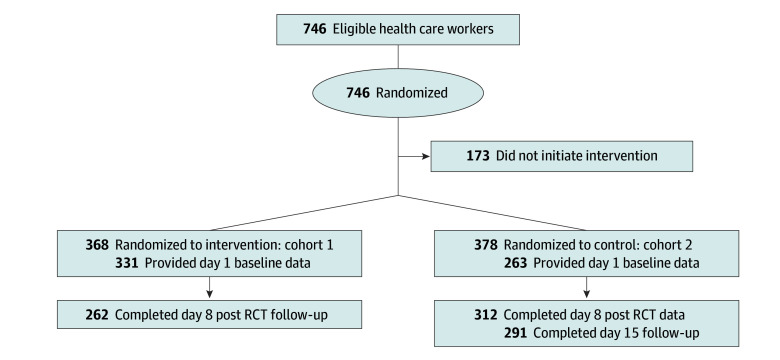
Patient Flowchart A total of 573 participants were analyzed. RCT indicates randomized clinical trial.

Enrollment and participation in the trial are shown in Figure 1. In cohort 1, 331 respondents (90%) initiated the intervention, with 262 (71%) in the post-RCT follow-up on day 8. In cohort 2, 312 respondents (83%) initiated the intervention, with 291 (77%) in the post-RCT follow-up on day 8. Table 1 displays the characteristics of the study population by cohort before the intervention. eTable 1 in Supplement 2 presents the numbers, mean (SD) values, and percent-concerning rates for both cohorts. Cohort populations were similar at baseline, with a total of 528 (89%) women and 93 (11%) men. Most of the participants were White (494 [83%]), and most of the roles were nurses (177 [30%]) or physicians (37 [6%]). Cohorts 1 and 2 had similar demographic characteristics at baseline. No adverse events were reported. Three of the 4 dimensions of HCW well-being exhibited good psychometric reliability pretest and posttest (ie, Cronbach α ≥0.80): emotional exhaustion (Cronbach α = 0.87 of 0.87), emotional thriving (Cronbach α = 0.82 of 0.87), emotional recovery (Cronbach α = 0.85 of 0.90), and work-life integration (Cronbach α = 0.33 of 0.46). eTable 2 in Supplement 2 presents work-life integration item-level results. Secondary analyses of the WLI items revealed that “Worked through a shift/day without any breaks” and “Changed personal/family plans because of work” improved. eTable 2 in Supplement 2 presents WLI item-level results.

**Table 1.** Characteristics of the Study Population Before Intervention

Characteristic	No. (%)				
	Cohort 1		Cohort 2		Control (n=312)^a^
	Enrollment (n=368)	Baseline (n=331)^b^	Enrollment (n=378)	Baseline (n=263)^b^	
Sex					
Male	41 (11.2)	37 (11.2)	36 (9.5)	26 (9.9)	26 (8.4)
Female	325 (88.8)	292 (88.8)	340 (90.2)	236 (90.1)	284 (91.3)
Other	NA	NA	1 (0.3)	NA	1 (0.3)
Race and ethnicity^c^					
African American	17 (4.6)	14 (4.3)	27 (7.2)	21 (8.0)	20 (6.5)
Asian	15 (4.1)	14 (4.3)	17 (4.5)	11 (4.2)	15 (4.8)
Hispanic	3 (0.8)	3 (0.9)	5 (1.3)	2 (0.8)	4 (1.3)
White	311 (85.0)	278 (84.5)	309 (82.2)	216 (82.4)	255 (82.3)
Other^d^	20 (5.5)	20 (6.1)	18 (4.8)	12 (4.6)	16 (5.2)
Age, y					
20-29	18 (5.0)	16 (5.0)	18 (4.8)	11 (4.2)	14 (4.5)
30-39	72 (20.2)	66 (20.6)	84 (22.5)	56 (21.5)	72 (23.4)
40-49	105 (29.4)	92 (28.8)	127 (34.0)	96 (36.9)	107 (34.7)
50-59	113 (31.7)	102 (31.9)	96 (25.7)	70 (26.9)	79 (25.6)
≥60	49 (13.7)	44 (13.8)	49 (13.1)	27 (10.4)	36 (11.7)
Discipline					
Behavior health	9 (2.5)	9 (2.7)	17 (4.5)	12 (4.6)	11 (3.6)
Medicine	68 (18.5)	64 (19.4)	64 (17.1)	46 (17.7)	53 (17.2)
Nursing	141 (38.4)	126 (38.2)	155 (41.4)	103 (39.6)	134 (43.5)
Public health	14 (3.8)	13 (3.9)	12 (3.2)	9 (34.6)	7 (2.3)
Other	135 (36.8)	118 (35.8)	126 (33.7)	90 (34.6)	103 (33.4)
Health care worker role					
Physician^e^	25 (6.9)	25 (7.7)	18 (4.9)	12 (4.7)	17 (22.8)
Nurse^f^	101 (27.9)	93 (28.5)	118 (31.9)	84 (32.7)	101 (32.0)
APP^g^	13 (3.6)	11 (3.4)	11 (3.0)	9 (3.5)	9 (6.6)
Other^h^	223 (61.6)	197 (60.4)	223 (60.3)	152 (59.1)	179 (38.2)

Abbreviations: APP, advance practice professional; NA, not applicable.

^a^ Cohort 2 served as control, providing day 8 post randomized clinical trial data without exposure to the intervention.

^b^ Baseline defined as day 1 before intervention.

^c^ The response choices for race and ethnicity presented to participants were to check all that apply in the “My race” category with the classifications American Indian or Alaska Native, Asian, Black or African American, Native Hawaiian or Other Pacific Islander, White, and Other.

^d^ No further breakdown of this category is available.

^e^ Physician includes attending, staff, fellow, and resident physician.

^f^ Nurse includes registered nurse, nurse manager, and charge nurse.

^g^ APP includes physician assistant and nurse practitioner.

^h^ Other roles include therapist (eg, respiratory, physical, occupational, and speech therapist), administrative support (eg, clerk, secretary, and receptionist), clinical support (eg, certified medical assistant, nurses’ aide), pharmacist, clinical social worker, manager, dietician/nutritionist, student, and others.

### WELL-B Efficacy

**Figure 2.**
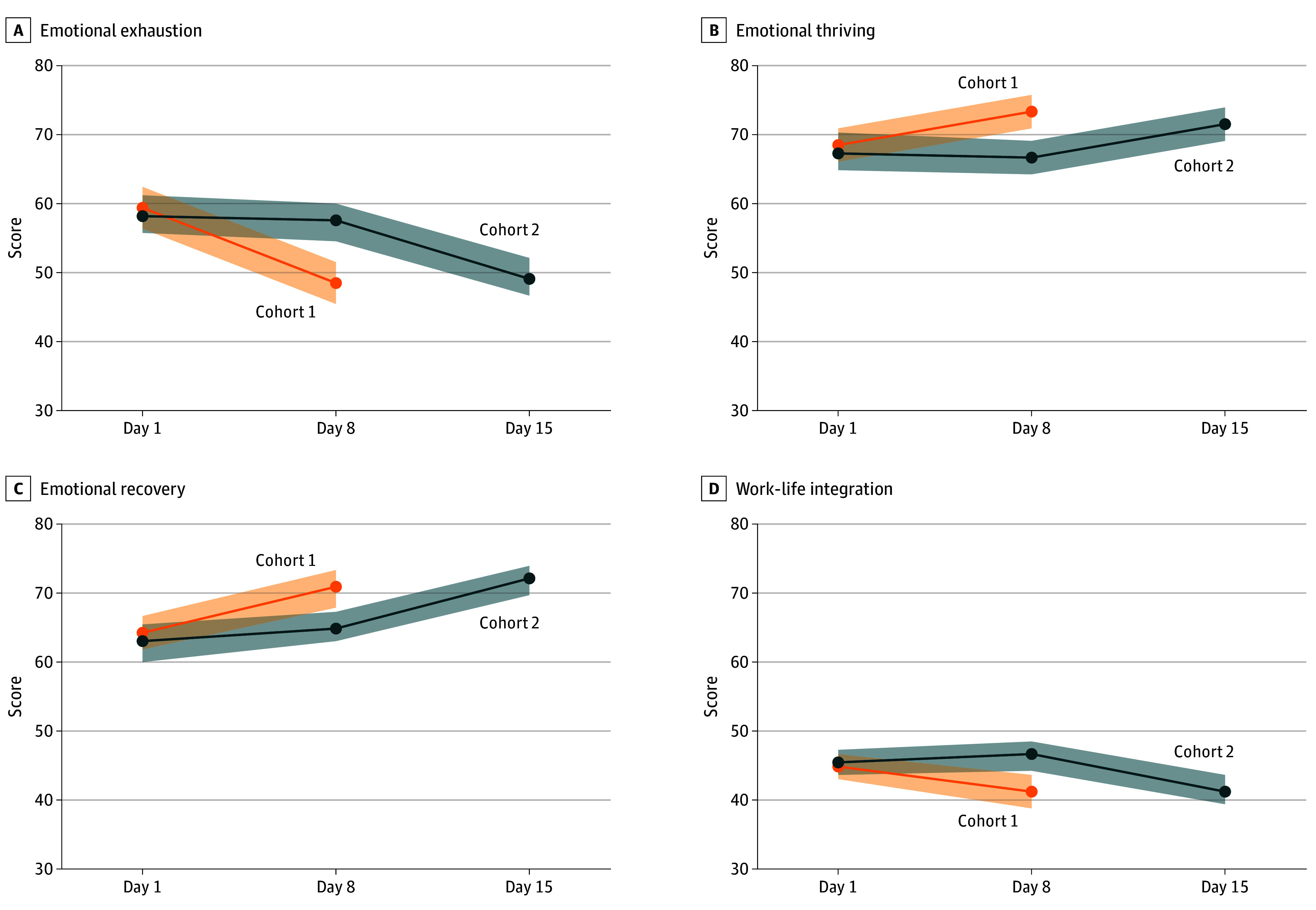
Mean Scores Following Intervention for Days 1, 8, and 15 by Cohort Scores on 100-point scales. Lines indicate means; shaded areas, 95% CIs.

On a 100-point scale, compared with cohort 2 (control), the WELL-B intervention in cohort 1 the WELL-B intervention in cohort 1 reduced emotional exhaustion (estimate: −9.0; 95% CI, −13.1 to −4.9; *P* < .001), improved emotional thriving (estimate: 6.6; 95% CI, 3.2-10.0; *P* < .001), emotional recovery (estimate: 5.5; 95% CI, 2.0-9.0; *P* = .002), and problematic work-life integration (estimate: −5.0; 95% CI, −8.2 to −1.9; *P* = .002) on day 8 (Table 2). After the RCT was completed, the WELL-B intervention in cohort 2 also reduced emotional exhaustion (estimate: −9.3; 95% CI, −11.3 to −7.2; *P* < .001) and problematic work-life integration (estimate: −5.8; 95% CI, −8.5 to −3.0; *P* < .001), and improved emotional thriving (estimate: 4.7; 95% CI, 2.8-6.6; *P* < .001) and emotional recovery (estimate: 5.8; 95% CI, 3.8-7.8; *P* < .001), when comparing measures on day 8 and day 15 (Figure 2).

**Table 2.** Intervention Effectiveness in Cohort 1 vs Cohort 2 on Day 8^a^

Outcome	Estimate (95% CI)	*P* value
Emotional exhaustion	−9.0 (−13.1 to −4.9)	<.001
Emotional thriving	6.6 (3.2 to 10.0)	<.001
Emotional recovery	5.5 (2.0 to 9.0)	.002
Work-life integration	−5.0 (−8.2 to −1.9)	.002

^a^ Same-day assessments using *t* tests compared 1-week postintervention for cohorts 1 and 2.

### WELL-B Magnitude of Change

After adjusting for baseline outcome measures, sex, race and ethnicity, age, health care worker role, and discipline, the linear regression model showed WELL-B improved day 8 emotional exhaustion (estimate: −9.6; 95% CI, −12.5 to −6.6; *P* < .001) compared with the control group for all 4 outcomes (Table 3; eTables 3-6 in Supplement 2). In the percent-concerning models, statistically significant improvements for all 4 outcomes were maintained, including a 16.8% reduction in the percent of participants reporting emotional exhaustion (Table 3).

**Table 3.** Effectiveness of WELL-B at Day 8 Using 100-Point Scale and Percent-Concerning Models^a^

Outcome	100-Point scale model		Percent-concerning model	
	Estimate (95% CI)	*P* value	Estimate (95% CI)	*P* value
Emotional exhaustion	−9.6 (−12.5 to −6.6)	<.001	−16.8 (−24.3 to −9.3)	<.001
Emotional thriving	3.6 (1.0 to 6.3)	.008	−8.9 (−16.9 to 0.8)	.03
Emotional recovery	3.1 (0.3 to 5.8)	.03	−9.2 (−17.3 to −1.1)	.03
Work-life integration	−6.2 (−9.5 to −2.8)	<.001	−8.7 (−16.9 to −0.5)	.04

Abbreviation: WELL-B, Well-Being Essentials for Learning Life-Balance.

^a^ Estimates for the WELL-B intervention (cohort 1) are compared with the control group (cohort 2). Linear regression models account for baseline outcome measures, sex, race and ethnicity (Asian, Black, Hispanic, White, and other), age, health care worker role, and discipline. One participant was not included in the model due to small counts for their sex designation (other).

For all 4 models, the measures at baseline were associated with each of the outcomes of interest on day 8 (emotional exhaustion estimate: 0.8; 95% CI, 0.7-0.8; *P* < .001; emotional thriving estimate: 0.8; 95% CI, 0.7-0.9; *P* < .001; emotional recovery estimate: 0.7; 95% CI, 0.6-0.8; *P* < .001; work-life integration estimate: 0.5; 95% CI, 0.4-0.6; *P* < .001). Additionally, male participants had lower levels of problematic work-life integration compared with females (estimate: −9.5; 95% CI, −14.9 to −4.0; *P* < .001) (eTable 6 in Supplement 2).

Participant evaluation of WELL-B on day 8 was positive across all 5 sessions, ranging from 90.3% to 98.0% reporting favorable impressions, and 94.4% to 97.3% reporting that the use of interesting and engaging content was appropriate (eTable 7 for participant evaluations and eAppendix for verbatim comments from participants in Supplement 2). Efficacy of the intervention was evidenced in an analysis of variance test comparing HCW completion of 5 vs 4 vs 3 or fewer sessions of WELL-B, finding no significant differences in day 8 emotional exhaustion (eTable 8 in Supplement 2). Emotional exhaustion improved within 8 days for the mostly live, mostly recorded, and mixed groups; there were no statistically significant differences comparing the types of sessions completed (eTable 9 in Supplement 2).

## Discussion

In this RCT of HCWs, WELL-B improved all 4 well-being outcomes of emotional exhaustion, emotional thriving, emotional recovery, work-life integration by day 8. Inserting meaningful opportunities for HCWs to engage in evidence-based positive psychology tools during continuing education proved to be feasible, available, and favorable across a variety of settings, roles, ages and demographic characteristics without any special equipment, personnel, prework, or postwork. Using the RAFT format to deliver evidence-based and engaging content allowed HCWs to complete the content, assessment, feedback and bite-sized interventions within each of the 5-hour sessions.

WELL-B was designed as a series of brief opportunities for the cultivation of positive emotions to alleviate emotional exhaustion, enhance emotional thriving, reinforce emotional recovery, and encourage better work-life integration behaviors. Participants used 10 minutes during a continuing education hour to reflect on their own well-being in a structured and evidence-based way. The largest effects were for emotional exhaustion and emotional recovery, meaning that participants were able to improve their emotional recovery while simultaneously alleviating their emotional exhaustion. They did not simply recover their well-being; it appears that, based on emotional recovery score, they enhanced their capacity to recover in the future.

The magnitude of well-being improvements caused by WELL-B between day 1 and day 8 is favorable compared with a meta-analysis of interventions to improve HCW well-being.^41^ WELL-B caused a 16.8% decrease in emotional exhaustion, which is greater than the 4.9% reduction in emotional exhaustion reported across 40 studies analyzed by West et al^41^ in their review of interventions to reduce burnout. Moreover, this 16.8% decrease in emotional exhaustion appears to be clinically meaningful because it is substantially larger than the 8.6% increase in emotional exhaustion attributed to the impact of the COVID-19 pandemic across 30 000 HCWs.^7^ The results also compare favorably with lengthier and more resource-dependent interventions intended to improve well-being and mental health, such as individualized coaching or meditation.^40,42,43,44^ The replicability of effects from the bite-sized positive psychology interventions delivered during WELL-B sessions is also critical to evaluating the present study results. Before this study, 2 other HCW RCTs using text message delivery of the interventions reported similar results in the same outcomes,^17,18,45^ and a growing number of cohort studies show this as well.^14,15,16,24^

WELL-B used the RAFT format to improve engagement through assessing, feeding back, and acting on well-being in real time during continuing education programs. The use of simple and engaging positive psychology activities was deliberate and, based on favorable ratings, resonated with participants. These structured opportunities to pause and reflect were sufficient to cause multidimensional improvements in well-being. Taken together, the magnitude, replicability,^14,15,17,18,45^ and biological pathway^19,20^ to explain recovery make WELL-B a useful and simple intervention for a weary workforce. Future studies should establish additional positive psychology intervention modules, assess sequencing effects for which modules work best and in what order, and determine the minimally effective dose so that the intervention is maximally accessible, available, feasible, and relevant.

Given the statistical equivalence of completing 5 vs 4 vs 3 or fewer sessions of WELL-B, future versions of WELL-B should explore 3- and 4-hour alternatives to further minimize the burden of well-being activities. Similarly, the statistical equivalence of using recordings vs attending live WELL-B sessions suggests that the use of recordings on demand would further facilitate usefulness and feasibility.

We found that participants completing 4 sessions were more likely to have higher baseline emotional exhaustion scores than those completing 5 or 3 or fewer sessions. This may be due to participants with higher emotional exhaustion scores having greater motivation to benefit from the intervention while being too busy to attend all 5 sessions. Prior research found individuals with higher emotional exhaustion were more motivated to do something to improve their well-being.^14^ Well-being intervention researchers are often concerned about a domain-specific conundrum: the participant needs at least enough well-being to initiate an intervention to improve well-being. Attrition in well-being interventions is particularly problematic for participants with high levels of burnout; however, WELL-B did not experience higher dropouts for participants with higher emotional exhaustion.

Well-being interventions based on positive psychology^46^ research may help alleviate some of the growing problems with HCW well-being. These problems became particularly salient during the unprecedented and prolonged upheavals associated with the COVID-19 pandemic.^7,47,48^ The robust improvements across 4 well-being outcomes demonstrated that WELL-B improves multiple aspects of well-being (ie, emotional exhaustion, emotional thriving, emotional recovery, and work-life integration) using brief online educational sessions. The effects did not differ significantly based on participating in live vs recorded sessions, nor did they differ significantly after adjusting for sex, race and ethnicity, age, HCW role, and discipline. There was not a demographic group for whom WELL-B was ineffective. Rather than target a demographic group, a future direction could look at the baseline well-being of each participant to match an intervention to a need. The use of latent profile analyses of well-being dimensions^49,50^ could be incorporated into future WELL-B applications to diagnose a well-being profile and treat each profile using interventions that are uniquely beneficial to that profile.

The HCW WELL-B user experience was positive, with more than 90% evaluating it favorably. Consistent with earlier RCTs of positive psychology interventions in HCWs,^17,18,45^ WELL-B showed that simple, low-resource interventions can cause robust improvements in individual HCW well-being. As of the dates of this study, WELL-B is available at no cost and on demand as a well-being intervention.^22^

### Concerns With Work-Life Integration

All 4 well-being outcomes were improved by day 8, but the improvement in work-life integration should be interpreted with caution. All scales exceeded the psychometric requirements for inclusion in this RCT, but an assessment of work-life integration responses before and after the intervention revealed a low level (0.33 of 0.46) of coherence among the work-life integration items. Generally, the α level of a scale should exceed 0.7 to be acceptable,^51^ 0.8 to be good, and 0.9 to be excellent. The psychometric shortfall of work-life integration was not expected, but it is possible that the concept of work-life integration is psychoactive and could introduce a measurement bias. Earlier RCTs of Web-Based Implementation for the Science of Enhancing Resilience (WISER)^17,18,45^ did not emphasize work-life integration as part of the intervention, but WELL-B was introduced to potential participants in presentations, flyers, and videos as an intervention to cultivate gratitude, work-life integration, self-compassion, awe, and group-level well-being. Given that most respondents reported dissatisfaction with their work-life integration, the spotlight WELL-B places on work-life integration may create intrusive thoughts that cause them to overthink their responses compared with participants in other well-being studies that do not specifically emphasize work-life integration. Answering questions about how often one eats or sleeps poorly, skips breaks, or gets home late after enrolling in a study to improve work-life integration may create a hyperfocus on work-life integration issues that introduces measurement bias. This is consistent with a previous RCT in which work-life integration improved during a wait-list control period during which participants answered work-life integration items multiple times (ie, repeatedly answering work-life integration items had the effect of an intervention to improve work-life integration).^18^ Nevertheless, 2 individual work-life integration items improved because of WELL-B (participants took more breaks and changed personal/family plans because of work less often) (eTable 4 in Supplement 2), and the other well-being scales improved monotonically. Further investigation of work-life integration interventions and metrics is warranted, and ideally, would not rely solely on the work-life integration scale to assess the effect. Anecdotally, we can report that the stand-alone work-life integration intervention on the CAWS website^52^ has generated similarly low α values; however, this has not occurred in any of the other 18 interventions that assess work-life integration but do not focus on work-life integration concepts (eg, humor, awe, and hope).

The use of a continuing education platform to deliver well-being resources was an innovative element of this RCT but does not suggest that any well-being resource delivered in this way will be similarly successful. WELL-B drew upon 15 years of effort in delivering bite-sized well-being interventions to HCWs, including 2 previous RCTs and dozens of iterations of pilot testing tool length, tool sequence, guided reflection instructions, and the gradual but substantial incorporation of participant feedback into the revisions. In other words, a well-being initiative that was successful in one pilot application should not be swiftly incorporated in continuing education as a delivery mechanism, without committing to substantial time and effort for development and revision.

### Limitations

This study should be viewed in light of its design limitations. Lack of long-term follow-up results for WELL-B was a consequence of the decision to improve recruitment by limiting participation to an 8-day intervention, without any prework or postwork. Recruitment of HCWs during and after the COVID-19 pandemic was challenging. Nevertheless, earlier RCTs using these bite-sized interventions have shown stable means at 6-month^17^ and 12-month^18,45^ follow-ups. In contrast to the effects of medication, which disappear on discontinuation, these positive psychology interventions promote well-being skills that endure. In line with other well-being behavioral intervention studies,^53,54,55,56,57,58^ we experienced noninitiation and attrition in both study cohorts, which may introduce selection bias. No measures of other well-being activities participants were engaging with (eg, yoga and exercise) were collected, but randomization to cohorts reduces the likelihood that the cohorts significantly differed in quantity or type of activities. In addition, the study participants were mostly White females, which reflects the workforce demographic characteristics in many large academic centers. Female HCWs report substantially worse well-being than their male counterparts,^59^ and female and racial and ethnic minority HCWs experience more mistreatment and discrimination by patients, families, and visitors in ways that deteriorate well-being.^60^ To address this, our models accounted for sex, race and ethnicity, age, HCW role, and discipline and showed that WELL-B still caused improvements across all the outcomes. Future studies can look to confirm that our findings are generalizable to settings with more diverse workforces.

## Conclusions

This RCT found that WELL-B significantly improved short-term HCW well-being by day 8 compared with a control, including a substantial reduction in emotional exhaustion. Improvements were consistent in models that controlled for demographic covariates. More than 90% of the participants evaluated WELL-B favorably. Although initiating any well-being intervention among busy HCWs can be a challenge, WELL-B used a continuing education format to pause and reflect on well-being through assessment, feedback, and engagement in a low-intensity positive psychology activity. This free intervention was brief, simple, highly valued, and evidence-based, and caused improvement within 8 days. The careful use of continuing education to provide well-being resources appears promising.

## Notes

Supplement 1.
Study Protocol

Supplement 2. **eTable 1.** Outcomes in Study Population **eTable 2.** Work-Life Integration Item-Level Results **eTable 3.** Emotional Exhaustion: Linear Estimates and 95% Confidence Intervals for Fixed Effects When Modeling WELL-B Intervention on Day 8 (n = 421) **eTable 4.** Emotional Thriving: Linear Estimates and 95% Confidence Intervals for Fixed Effects When Modeling WELL-B Intervention on Day 8 (n = 420) **eTable 5.** Emotional Recovery: Linear Estimates and 95% Confidence Intervals for Fixed Effects When Modeling WELL-B Intervention on Day 8 (n = 420) **eTable 6.** Problematic Work-Life Integration: Linear Estimates and 95% Confidence Intervals for Fixed Effects When Modeling WELL-B Intervention on Day 8 (n = 419) **eTable 7.** WELL-B Participant Evaluations **eTable 8.** Participant Completion of 5 vs 4 vs 3 or Fewer Sessions of WELL-B **eTable 9.** Live vs Recorded Sessions **eAppendix.** Verbatim Comments of RCT Participants

Supplement 3.
Data Sharing Statement
