# Supplementary material for: Well-Being Outcomes of Health Care Workers After a 5-Hour Continuing Education Intervention: The WELL-B Randomized Clinical Trial
Source: JAMA Netw Open. 2024 Sep 19;7(9):e2434362. doi: 10.1001/jamanetworkopen.2024.34362 (PMC11413716; doi:10.1001/jamanetworkopen.2024.34362)
Supplement: Supplement 1. — Study Protocol [file jamanetwopen-e2434362-s001.pdf]

Supplemental for

Well-being Outcomes of Healthcare Workers after a 5 Hour Continuing Education Intervention: The WELL-B Randomized Clinical Trial

ClinialTrials.Gov Identifier:
NCT05636072

Principal Investigator:
J. Bryan Sexton, PhD
Associate Professor of Psychiatry, Duke University
Co-Investigator:
K. Carrie Adair, PhD
Assistant Director of Well-being and Research
Duke Center for the Advancement of Well-being Science (CAWS)
Duke University Health System
https://caws.dukehealth.org/
linkedin.com/in/wellb

Phone:
410-746-3559
Email:
Bryan.Sexton@duke.edu

Funded by:
Health Resources and Services Administration (5 U3NHP45396-02-00, PI: JBS)

Original Trial Protocol and Statistical Analysis Plan

Purpose of the Study

Objectives & hypotheses to be tested

The objective of this study is to determine how a Well-being Collaborative web-based seminar series, and online resilience tools, in full or in part, will impact individual well-being. Efficacy of the resilience sessions and tools will be assessed using psychometrically validated survey scales of burnout, depression, happiness, work life balance, disruptive behaviors, emotional thriving, emotional recovery, optimism and state-based anxiety.

The primary hypothesis is that the web-based series and tools will improve individual measures of burnout, depression, happiness, work life balance, disruptive behaviors, emotional thriving, emotional recovery, optimism and state-based anxiety when comparing pre-intervention and post-intervention matched data. We further hypothesize that individual improvements will have a spillover effect on aspects of the shared work environments including disruptive behaviors.

Background & Significance

Should support the scientific aims of the research

Burnout syndrome is a work-related psychopathology comprised of emotional exhaustion, cynicism and inefficacy, which occurs in professionals who do “people work.”1,2 Burnout develops form chronic exposure to interpersonal stressors – such as frequent interactions with individuals plagued by complex physical, psychological and social problems.1,2 Burnout is also perpetuated by unsupportive or inadequate work environments.3 Healthcare workers are uniquely vulnerable to burnout as the complex physical crises of patients are often accompanied by equally complex psychosocial and spiritual crises of the individuals and their families. Burnout has been associated with low job satisfaction, absenteeism, poor staff retention, low staff morale, poor performance, disturbed sleep, poor health outcomes and even higher all-cause mortality.2,4-8

The prevalence and severity of burnout syndrome in healthcare workers is under-recognized as a significant factor affecting the quality and safety of care. The prevalence of burnout in healthcare workers ranges from 27-86%.9-13 More alarming is the correlation between burnout and sub-optimal patient care and medical errors.10,14 Despite the need for interventions to reduce healthcare worker burnout, few exist.15

In recent assessments of unit culture across the Duke University Health System, high burnout has paralleled poor patient safety and teamwork norms across 440 clinical areas. Not surprisingly, this pattern is also seen in other health systems the Duke Center for Healthcare Safety and Quality collaborates with across the country. We hypothesize that an intervention focused on resilience training will improve measures of personal well-being (e.g burnout, depressive symptoms, happiness) and improve teamwork and safety culture. With the hopes of validating this resilience intervention for use elsewhere, we chose to conduct a formal research study. (see Research Summary document for references)

Design & Procedures

Describe the study, providing detail regarding the study intervention (drug, device, physical procedures, manipulation of the subject or the subject’s environment, etc.). Discuss justifications for placebo control, discontinuation or delay of standard therapies, and washout periods if applicable. Identify procedures, tests and interventions performed exclusively for research purposes or more frequently than standard of care. Include alternative therapies, concurrent therapies discontinued per protocol, risk benefit ratio, and use of tissue/specimens. Discuss monitoring during washout periods if applicable. Include brief description of follow-up, if any.

This is a study of individuals 18 and over who voluntarily enroll in a web-based well-being series and participate in surveys to assess the effect of the intervention. The intervention will not involve patients, tissues/specimens, drugs, devices, or placebo controls.

Resilience Collaborative Web-based Series

The Resilience Collaborative Web-based series is a condensed version of the two day “Enhancing Caregiver Resilience: Burnout & Quality Improvement Course” offered by the Duke Center for Healthcare Safety and Quality since 2009. The webinar series is commonly referred to as WISER, short for Web-based Implementation for the Science of Enhancing Resilience. WISER is offered in three formats, full, condensed, and 4-day. The full format of WISER consists of either 12 or 24 webinars, approximately 60 minutes in length each. The condensed format of WISER consists of 10 minute pre-recorded videos using the same content, just offered in smaller chunks. For work settings that afford little logistical opportunity to watch an hour-long video, like NICUs or busy primary care

practices, the condensed format of WISER is recommended. For work settings that have continuing education windows or more logistical flexibility, the full format of WISER is recommended. The most recent version, the 4-day format of WISER, involves 4 consecutive days of 1-hour live or recorded webinars.

WISER is also offered via a randomized control trial design to assess the 5-day format. This format, called WELL-B, introduces participants to a subset of topics in the full WISER format- specifically four of the WISER tools, 1 per day (i.e., Work-life balance, Gratitude, Self-compassion, and Awe). This RCT will occur in the context of a 5 day webinar training. Enrollees will be randomized during electronic informed consent in Qualtrics to either cohort 1 (M-Th) or cohort 2, which occurs the following week (M-Th) via webinar. For those assigned to cohort 1, assessments will occur the first day of the training and day 8 (1-week after the training) as well as 1, 6, and 12 month follow-ups. For those assigned to cohort 2, assessments will occur on their first day of training (day 8 of cohort 1), 1 week after the training, and at 1, 6, and 12 month follow-ups. The RCT comparison will assess tool efficacy between the cohort 1 (week 1) and cohort 2 (week 2). Follow-ups will examine the long term efficacy of the tools.

Another randomized control design of WISER will specifically look at the "Looking Forward" WISER tool.

The Looking Forward Tool asks participants to "Think about something you hope to experience or are looking forward to 3 years from now. ..." and describe what you are looking forward to in 3-4 sentences. Participants will be recruited by the same current method: learning about the tools in a continuing education offering or newsletter, and enrolling through a link on our website. Participants will complete baseline measures and be randomized to either begin the tool immediately (cohort 1), or wait 4 days to begin (cohort 2). Both groups will be assessed on day 4 (waitlist group/cohort 2 will be assessed before they begin the tool). Cohort 1 will continue on with the tool 3 more days; once initiated, the tool is 7 days total for both groups. The RCT comparison will assess treatment efficacy between 4 days of the tool (cohort 1) to the waitlist group (cohort 2). Both groups will be assessed at 1 and 6 month follow-ups for long term efficacy.

We also offer WISER through an observational arm, whereby investigators seek to optimize the number and sequence of WISER modules. The observational arm is intended to help us understand which components of WISER have the biggest and most enduring impact on well-being across different healthcare delivery settings, demographic groups, as well as which modules should be offered earlier in the series so as to maximize participation and improvement in well-being.

The full, condensed, and 4-day versions of WISER were created according to evidence-based principles of adult learning, combining educational material with practice-based learning. We demonstrate the need using evidence of the prevalence and severity of burnout, then the evidence regarding the efficacy of the specific tool being covered, then present the tool for practice-based learning.

Module content WISER condensed: The condensed version of WISER consists of pre-recorded videos, each under 10 minutes in length, playable during use of the tool. The videos are highly refined to be engaging, evidence based, and practical as a launch to the subsequent reflective activity using very brief and enjoyable applications of the module content.

Module 1. Thematic Introduction prevalence of burnout, evidence of enhancing resilience and overview of the next 6 modules.

Module 2. Gratitude: This module provides a structured opportunity to learn about the science behind gratitude tools and express gratitude in general and toward others through a guided gratitude letter writing exercise\. Through expressing gratitude, we learn more about our vital connections to others, often in surprising and meaningful ways, and we see improvements in well-being, sleep quality and depression.

Module 3. Three Good Things: This module introduces a structured opportunity to cultivate positive experiences by reflecting on them for a couple of minutes within two hours of sleep onset (daily email/SMS sent to each participant for 2 weeks). By savoring good moments from earlier that day just before sleep onset, we see improvements in well-being, sleep quality, relationships, work-life balance, and depression.

Module 4. Awe: This module provides a structured opportunity to learn about and experience the benefits of awe and wonder through a series of visually and conceptually stunning images, followed by an exercise to recount in detail one of their own experiences of awe and a daily "moment of awe" log. Participants are encouraged to share moments of awe with others and elicit their moments of awe as an opportunity to connect with others. When we experience awe, our sense of time expands, we are kinder to others, and we prefer experiences over material things. By cultivating awe we see improvements in life satisfaction, kindness, and sense that we have more time to do the important things in life.

We give participants the option to submit photos to us, via qualtrics, that evoke or represent a sense of "awe" to them. We will not share these photos with other participants and the photos will be saved on a secure server. The photos provide a visual focus for the awe exercise that can also be completed using a memory if the participant chooses.

Module 5Identifying and Using Signature Strengths: This module uses a strengths-finder tool to examine what you do well, and sends daily prompts for one week to elicit how participants will be deliberate about using their strengths in new and more frequent ways to address their challenges. When you use your strengths personally and professionally, you feel engaged and invigorated, and you feel like what you are doing has more meaning to you personally. By deliberately choosing to do what we already do well, we see improvements in engagement, happiness and depression.

Module 6. Random Acts of Kindness: This module uses a nightly log of acts of kindness for 8 days. By committing to random acts of kindness and savoring them later through the use of a log, participants experience temporary but noticeable increases in subjective well-being.

Module 7. Relationship Resilience/ One Good Chat: This module uses 8 days of brief reflections on warm and uplifting interactions as for a strategy to enhancing well-being by savoring brief positive interactions with others.

Module 8. Looking Forward Module. This module asks participants to write reflections about what they are looking forward to at various point in their life - the near future and many years down the road. Research shows that people spend a considerable time envisioning future prospects, but often times this thinking can include worries or fears, rather than what they are looking forward to. This module is to help individuals envision positive events in the future to potentially evoke a feeling of hope rather than worry.

Module 9. Positive Emotion Tracking. This module asks participants to rate the frequency and/or intensity of their emotions, including various positive emotions. Research shows that people with a ratio at or above approximately 3

positive emotions to every 1 negative emotion are more likely to be thriving in their lives. By reporting on their emotions over time, we expect participants to become more aware of their emotions and to engage in behavior that increases their positive emotions, and potentially decreases their experience of negative emotions.

Module 10. Mindfulness Moment. Research has shown that mindfulness meditation confers a variety of salutary effects. This tool will guide participants through a brief mindfulness break (between 1-3 minutes) in which they will focus on the present moment.

Module 11. One Door Closes, Another Opens. Positive reappraisal of difficult experiences is a cognitive mechanism for coping and wellbeing. This tool asks participants to briefly write about any benefits (in the short or long term) that may have resulted from an experience which at first appeared entirely negative.

Module 12. Three Funny Things. Similar in structure to the Three Good Things tool, Three Funny Things asks participants to record funny things that happen each day for 8 days. Research in other labs has identified that doing so improves well-being. It is thought that by being asked to recall funny things prompts the participant to view ongoing experiences through a more humorous lens.

Module 13. The Sleep Tool. This tool asks participants to record each morning for 8 days one's sleep quantity and quality. Evidence-based sleep strategies are prompted each day, such as use of screens, naps, caffeine, and exposure to blue light from sunrises and sunsets. We've found in our research that reflecting on these behaviors appears to improve them over time.

Module 14. Interest Tool. The interest tool was built off research linking curiosity to well-being. It asks participants "What is interesting to you lately?" and to write about it for a couple of minutes. The tool is repeated 4 times over the course of a week.

Module 16. Feedback Tool. This tool will allow participants to report on and track the feedback that they are providing to others, the amount of feedback that they receive, and the amount of feedback that they view occurring between their colleagues.

Module 17. Prioritizing Positivity Tool. In this tool participants are asked to set an intention or goal to participate in activities that give them joy or other positive emotions. The tool encourages achievement in setting this goal and making it a habit by selecting activities that are easy to fit into participants' schedules. This tool is built off of research by Dr. Catalino that finds prioritizing positive experiences is an effective route to happiness.

Module 18. Your Burnout Story (Expressive Writing) Tool. Inspired by research on the benefits of expressive writing to process difficult emotions, this tool asks participants to write the story of any experiences they've had with burnout. The idea is to process the experience of burnout, reflect on the factors that lead up to it, and provided relief from it, and take lessons from the writing process.

Module 19. Self-compassion Tool. This tool asks participants to reflect on a personal experience they found difficult and that they were overly self-critical about. Next, participants are asked to imagine they were speaking to a good friend about the incident, and to discuss it in a more self-compassionate light.

Module 20. Cultivating Forgiveness Tool. This tool asks participants to reflect on a personal experience where they were wronged and would now like to cultivate forgiveness. Participants are asked to spend 5-7 minutes writing a letter of forgiveness.

Module 21. Pride Tool. The pride tool was built off research linking pride in one's accomplishments to well-being. It asks participants "What is an example of something you deem to be "Fridge-worthy" as in an accomplishment that you would have put on the refrigerator in your kitchen?" and to write about it for a couple of minutes on the screen that has a background image of a large refrigerator. The tool is repeated one week later.

Module 21. Serenity Tool. The serenity tool was built off research linking the positive emotion of feeling at peace with the world around you to well-being. It asks participants to choose a moment when they have 2 minutes without interruption, for example in the car before driving home from work, and to commit to doing nothing for those two minutes. The tool is repeated daily for 8 days.

Module 22. Cultivating Inspiration Tool. This tool asks participants to reflect on people, images, and events that fill them with a positive desire to do good in the world. Participants are asked to spend 5-7 minutes writing a letter of inspiration first, and then again 1 week later.

Module 23. Cultivating Love Tool. This tool asks participants to reflect on people that recharge their batteries and to spend 5-7 minutes writing a letter of what their relationship means to them in detail, then are asked to do it again 1 week later.

Module 24. The Work-life Balance Tool. This tool asks participants to reflect on work-life balance behaviors and activities for 4 days. Behaviors include exercise, spending time with a friend or loved one, spending time in nature, etc. We've found in our research that reflecting on these behaviors appears to improve work-life balance over time.

Module 25. The Grief Tool. This tool asks participants to reflect on a loss they have experienced (e.g., a loved one, a pet, a job, a divorce, etc.). They briefly write about the loss, then engage in a letter-writing activity in which they write from the perspective of a loved one offering them support.

Module 26. The Coaching Tool. This tool asks participants to pause and reflect on their own well-being as well as the well-being of their teams. Participants engage in 5 coaching interactions and complete reflective exercises.

To maximize convenience for research subjects, the recordings will be available for individual viewing over the web and can be viewed individually or shared for group viewings depending on staff and provider availability.

Module content WISER full version: The full version of WISER consists of 12 videos (up to 24 total if the participant chooses to continue another year), approximately 55 minutes in length each, given over 12 months. The brief version of WISER includes videos that are 2-10 minutes in length, and contains the most important elements of the full videos. The videos are highly refined to be engaging, evidence based, and practical as a launch to the following fourteen days of behavioral modification using very brief and enjoyable applications of the module content.

The topics for each WISER full format web-based session, in chronological order, include: Prevalence & Severity of Burnout: Workforce Resilience as Care Quality, Enhancing Resilience: The Science and Practice of Gratitude, Relationship Resilience: The Science of How Other People Matter, Enhancing Resilience: Three Good Things, Enhancing Resilience: Practicing Safe Stress and the Science of Sleep, Psychological Safety: The Predictive Power of Feeling Supported When Things Go Wrong, The Science of Mindfulness, Health Care Worker Resilience, Work Life Integration, and Burnout, Collaboration vs. Dealing with Difficult Colleagues: Assessing, Understanding and Improving Teamwork in a Clinical Area Near You, Science of Wow: Cultivating Awe and Wonder as a Resilience Strategy, Positive WalkRounds: Leader Rounding to Identify What is Going Well – Links to Quality, Culture and Workforce Resilience, Enhancing Resilience: Survival of the Kindest, If Culture Eats Strategy for Lunch, Burnout Eats Culture for Breakfast: New Evidence About the Contagion Effect of Burnout and Impact on Safety Culture, The Funny Thing about Resilience: Evidence for Humor, Improvement Readiness in Healthcare: Introducing a Metric that assesses the capacity within a work setting to start and sustain quality improvement efforts, Coping with Change and the Neuroscience of Hope, Signature Strengths at Work, Second Victim of Harm: Coping After Things Go Wrong, The Surprisingly Robust Science of Self Compassion, Institutional Resources vs. Individual Resources as Solutions for Healthcare Worker Well-Being, Overview of Team Training, Tools, Techniques and Integration into Existing Infrastructure, Absence of Burnout is not the same as Thriving: Moving from Deficit Metrics to Flourishing Metrics for Healthcare Workers, Patient Safety Leadership WalkRounds: Links Safety Culture, Burnout and Workforce Well-Being, The Pursuit of Happiness: Methods vs. Mythology. To maximize convenience for research subjects, the recordings will be shared with unit managers who will coordinate multiple viewings for their clinical areas, based on staff and provider availability.

Resilience Collaborative Survey

A pre-intervention survey will be administered prior to the first webinar and post-intervention surveys will be administered following the last webinar and again 1, 6, 12, 18, and 24 months later. The pre and post surveys will be nearly identical and consist of demographic variables (e.g. healthcare system, clinical area, position, years of experience, gender, and shift), a 5-item version of the Emotional Exhaustion dimension of the Maslach Burnout Inventory[1], the Center for Epidemiological Studies Depression Scale-10-item version (CES-D10)[18], the Subjective Happiness Scale[19], the Work-Life Activities and Behaviors Questionnaire (James Pennebaker, PhD), Optimism (LOT-R)[20], Anxiety (GAD-7)[21]and Emotional Thriving and Emotional Recovery.[22]Additional unpublished spiritual climate items, turnover intention items, and items pertaining to vacation time, will be asked as well. The pre- and post- intervention surveys will take participants approximately 15 minutes, or less, to complete.

In addition to the pre- and post- intervention surveys, brief peri-intervention surveys will be administered during each webinar. The purpose of the peri-intervention surveys are to ascertain whether individuals absorbed the main concepts of the webinar and to evaluate the efficacy of specific interventions shared during the intervention (e.g. the three good things exercise).

A separate continuing education (CE) survey to evaluate participant attendance and course content in order to award participants continuing education credit will be sent at the end of the module content (prior to refresher webinars). Credit will be offered and awarded in the form of IACET Continuing Education Units (CEUs) to all participants who complete the study. The names of participants who complete the CE survey will be shared with the CEU office.

CEUs may be considered an incentive for attending the webinars, however there is no requirement that healthcare workers participate in the research component of this study to be awarded CEU credit.

Participation will be voluntary and surveys will be administered electronically to maintain confidentiality. Qualtrics Survey Research Suite will be used to distribute, collect and store survey data. Enrollment links that contain the pre-intervention survey questions are available on our website, newsletter, and during continuing education sessions as part of the slides. Those individuals who wish to participate in the research will complete the consent form which is followed by the pre-intervention survey. Those who wish only to participate in the continuing education credit component will be asked for their email address, and these records used solely for the purpose of awarding continuing education at the end of the series. Those who do not wish to participate in either the research or the continuing education opportunity will not be asked to enter any additional information, and thus the study team will have no record of identifying information for these individuals. Research subjects who initially consent to the research study may request to have their email address removed from our study list serve at any time, in response to any electronically mailed survey. There is an "opt out" link in every survey sent to participants. Longitudinal survey data will be collected from each participant and matched using a unique study number created for each participant by the Qualtrics survey software. The pre-intervention survey is the only survey which will ask respondents for a unique identifier (email address), as this is necessary for follow up. Subsequent survey data files will only contain the unique study numbers of each participant. The data from the pre-intervention survey will be removed from the Qualtrics database within 3 months of collection and study personnel will remove the email address identifiers from the dataset at this time and prior to analysis. All data files, once removed from Qualtrics, will be stored on the encrypted study computers for at least 6 years following the completion of the study. Survey results, once matched, will be analyzed in aggregate by clinical area or healthcare worker group. Clinical areas or healthcare worker groups with less than 5 respondents will be excluded from the analysis to ensure responses of individual participants cannot be identified. Participants may choose not to respond to any of the surveys or specific survey questions, or may withdraw from the study at any time.

Individual Module Survey

The observational arm of this research does not use the entire pre-selected set and sequence of modules, but rather allows tools to be accessed individually. Unlike the full and condensed versions of WISER, this consists of briefer surveys pre and 1, 6, and 12 months post, and exclude spiritual climate items (taking participants approximately 8-10 minutes to complete).

Selection of Subjects

List inclusion/exclusion criteria and how subjects will be identified.

Adults 18 and over are considered eligible for the study. A sub-sample will include employees (including but not limited to frontline caregivers, support staff and management) of the Duke University Health System, as well as external health systems. All participants will be invited to participate in one or more of the online tools. We will allow the participation of any clinical areas/healthcare worker groups whose leaders express interest in building resilience.

We are targeting healthcare workers for recruitment in the study, but adults 18+ are eligible to participate in any of these tools. Identification as a healthcare worker or not is part of data collection in every tool.

Adults who are not proficient in English, do not have basic computer skills, or who have prohibitive vision or hearing disabilities will be excluded from this study as they will be incapable of full participation in the intervention and/or the survey process. However, given the nature of their occupations we anticipate few, if any exclusions.

Subject Recruitment and Compensation

Describe recruitment procedures, including who will introduce the study to potential subjects. Describe how you will ensure that subject selection is equitable and all relevant demographic groups have access to study participation (per 45 CFR 46.111(a) (3)). Include information about approximately how many DUHS subjects will be recruited. If subjects are to be compensated, provide specific prorated amounts to be provided for expenses such as travel and/or lost wages, and/or for inducement to participate.

Potential participants may learn about the links to the WISER tools at a talk or workshop given by the researchers. They may also learn about them from word of mouth of someone attending one of these talks, or the website for the Duke Center for the Advancement of Well-being Science (https://caws.dukehealth.org). Talks include grand rounds, formal presentations for continuing education, and virtual presentations for continuing education credit. At Duke North, for example, this could be at Duke 2100, or for those logging in to watch it virtually while it's being given at Duke North 2100.

For the full WISER RCTs, managers and directors of targeted clinical areas, as described above, will be contacted via email and asked to attend the introductory webinar for managers and directors. Managers and directors may voluntarily spread the word about WISER to their work settings or in response to a follow up email after the introductory webinar.

We estimate that approximately 500,000 adults (18 or older as confirmed by their informed consent statement that they are 18 or older) will participate in the study. 10,000 participants are expected to work at Duke University Health System.

Attrition is a concern in behavioral interventions such as WISER. We will address attrition in several ways. (1) By offering an intervention that is fun, practical, and both personally and professionally transformative. (2) Minimizing any time commitment for participants, which is why we have shortened and edited the webinar/videos in a parsimonious yet appealing manner to allow for viewing at e.g., shift change. (3) Participants can claim continuous education credits for completing the modules. (4) Participants will receive personalized confidential feedback of their well-being metrics.

Consent Process

Complete the consent section in the iRIS Submission Form.

Subject's Capacity to Give Legally Effective Consent

If subjects who do not have the capacity to give legally effective consent are included, describe how diminished capacity will be assessed. Will a periodic reassessment occur? If so, when? Will the subject be consented if the decisional capacity improves?

Data Analysis & Statistical Considerations

Describe endpoints and power calculations. Provide a detailed description of how study data will be analyzed, including statistical methods used, and how ineligible subjects will be handled and which subjects will be included for analysis. Include planned sample size justification. Provide estimated time to target accrual and accrual rate. Describe interim analysis including plans to stop accrual during monitoring. Phase I studies, include dose escalation schema and criteria for dose escalation with definition of MTD and DLT.

There are approximately 5000 health care workers working within fewer than 300 clinical areas nested within the participating healthcare systems. Multi-level modeling will be used to account for nesting, while descriptive statistics, GLM, and T-tests will be used to explore distributions and relationships. The three good things logs will be analyzed using linguistic analysis software.

Data & Safety Monitoring

Summarize safety concerns, and describe the methods to monitor research subjects and their data to ensure their safety, including who will monitor the data, and the frequency of such monitoring. If a data monitoring committee will be used, describe its operation, including stopping rules and frequency of review, and if it is independent of the sponsor (per 45 CFR 46.111(a) (6)).

Electronic survey data will be collected and stored using Qualtrics Survey Research Suite. Qualtrics has SAS 70 Certification and meets the Health Insurance Portability and Accountability Act (HIPAA) privacy standards. All Qualtrics accounts are hidden behind passwords and all data is protected with real-time data replication.

Documentation of the date and time of the consent e-signature is required. Through Qualtrics, there is a timing feature that collects the date and time and length of time each participant spends on a part of the survey. This time stamp is collected through Qualtrics as part of the pre-survey during the portion of the pre-survey used to indicate consent. Time stamps are collected using Mountain time, due to the fact that Qualtrics has its headquarters in Provo, Utah.

Data will be analyzed using IBM SPSS Statistics 20 on the study computers, which are protected with PGP Whole Disk Encryption from Symantec. Data will also be stored on these computers, and backed up in a private folder on a duke file server located in FITZ East. A deidentified dataset will be saved in a folder on a Duke University School of Nursing Secure Electronic Data (SED) folder: s:\\SED\. The folder will only be accessible by Duke personnel on the study. PGP Whole Disk Encryption from Symantec is built with high-performance Hybrid Cryptographic Optimizer (HCO) technology with Advanced Encryption Standard (AES). PGP Whole Disk Encryption is Federal Information Processing Standards (FIPS) 140-2 validated, CESG Assisted Products Scheme (CAPS) approved, Defence Infosec Product Co-Operation Group (DIPCOG) approved, and has achieved Common Criteria Evaluation Assurance Level (CC EAL) 4+ certification.

No data will be transferred from Duke or shared with external collaborators via Duke's institutional secure messaging tools without a Data Transfer Agreement in place with the receiving institution and without an approved amendment of the IRB protocol for that transfer.

Dr. Bryan Sexton will be responsible for managing the data and software described above. Email: bryan.sexton@duke.edu, telephone: (410) 746-3559.
