# Supplementary material for: Well-Being Outcomes of Health Care Workers After a 5-Hour Continuing Education Intervention: The WELL-B Randomized Clinical Trial
Source: JAMA Netw Open. 2024 Sep 19;7(9):e2434362. doi: 10.1001/jamanetworkopen.2024.34362 (PMC11413716; doi:10.1001/jamanetworkopen.2024.34362)
Supplement: Supplement 2. — eTable 1. Outcomes in Study Population eTable 2. Work-Life Integration Item-Level Results eTable 3. Emotional Exhaustion: Linear Estimates and 95% Confidence Intervals for Fixed Effects When Modeling WELL-B Intervention on Day 8 (n = 421) eTable 4. Emotional Thriving: Linear Estimates and 95% Confidence Intervals for Fixed Effects When Modeling WELL-B Intervention on Day 8 (n = 420) eTable 5. Emotional Recovery: Linear Estimates and 95% Confidence Intervals for Fixed Effects When Modeling WELL-B Intervention on Day 8 (n = 420) eTable 6. Problematic Work-Life Integration: Linear Estimates and 95% Confidence Intervals for Fixed Effects When Modeling WELL-B Intervention on Day 8 (n = 419) eTable 7. WELL-B Participant Evaluations eTable 8. Participant Completion of 5 vs 4 vs 3 or Fewer Sessions of WELL-B eTable 9. Live vs Recorded Sessions eAppendix. Verbatim Comments of RCT Participants [file jamanetwopen-e2434362-s002.pdf]

Supplementary Online Content

Sexton JB, Adair KC. Well-being outcomes of health care workers after a 5-hour continuing education intervention: the WELL-B randomized clinical trial. *JAMA Netw Open*. 2024;7(9):e2434362. doi:10.1001/jamanetworkopen.2024.34362

eTable 1. Outcomes in Study Population

eTable 2. Work-Life Integration Item-Level Results

eTable 3. Emotional Exhaustion: Linear Estimates and 95% Confidence Intervals for Fixed Effects When Modeling WELL-B Intervention on Day 8 (n=421)

eTable 4. Emotional Thriving: Linear Estimates and 95% Confidence Intervals for Fixed Effects When Modeling WELL-B Intervention on Day 8 (n=420)

eTable 5. Emotional Recovery: Linear Estimates and 95% Confidence Intervals for Fixed Effects When Modeling WELL-B Intervention on Day 8 (n=420)

eTable 6. Problematic Work-Life Integration: Linear Estimates and 95% Confidence Intervals for Fixed Effects When Modeling WELL-B Intervention on Day 8 (n=419)

eTable 7. WELL-B Participant Evaluations

eTable 8. Participant Completion of 5 vs 4 vs 3 or Fewer Sessions of WELL-B

eTable 9. Live vs Recorded Sessions

eAppendix. Verbatim Comments of RCT Participants

This supplementary material has been provided by the authors to give readers additional information about their work.

© 2024 Sexton JB et al. *JAMA Network Open.*

eTable 1. Outcomes in Study Population

Outcome	Cohort 1					Cohort 2					
	Enrollment		Baseline [*]			Enrollment		Baseline[*]		Control [**]	
**100-point scale (n, mean (SD))**											
Emotional Exhaustion			331	59.1 (26.2)				263	58.1 (24.1)	312	57.1 (24.9)
Emotional Thriving			331	68.3 (20.5)				262	67.1 (21.6)	312	66.3 (20.7)
Emotional Recovery			331	64.4 (23.0)				262	63.0 (21.4)	312	65.3 (20.9)
Work-Life Integration			330	44.8 (16.9)				262	45.6 (16.5)	312	46.7 (18.1)
**Percent concerning rate [8]**											
Emotional Exhaustion			68.0					68.1		66.3	
Emotional Thriving			50.2					55.3		55.1	
Emotional Recovery			54.1					58.0		55.4	
Work-Life Integration			75.2					78.6		77.9	

[1] Physician includes Attending, Staff, Fellow, and resident Physician. [2] Nurse includes Registered Nurse, Nurse Manager, and Charge Nurse. [3] Advance Practice Provider (APP) includes Physician Assistant and Nurse Practitioner. [4] Other roles include Therapist (e.g., Respiratory, Physical, Occupational, and Speech Therapist), Administrative Support (e.g., Clerk, Secretary, and Receptionist), Clinical Support (e.g., CMA, Nurses Aid), Pharmacist, Clinical Social Worker, Manager, Dietician/Nutritionist, Student, and others. [5] Surgical specialty includes Anesthesiology, Obstetrics & Gynecology, and Surgery. [6] High intensity medical care specialty includes Emergency Medicine, Critical Care Medicine, and NICU. [7] Other specialties include Family Practice, Internal Medicine, Neurology, Physical Medicine & Rehabilitation, Preventive Medicine, Psychiatry, Radiology, and others. [8] Percent concerning rates were calculated using previously published thresholds. [*] Baseline defined as Day 1 prior to intervention. [**] Cohort 2 served as control, providing Day 8 post RCT data without exposure to the intervention. [†] Categories with ≤ 5 individuals are not reported in order to protect participant privacy. Data may not add up to 100% due to missing data.

© 2024 Sexton JB et al. *JAMA Network Open.*

eTable 2. Work-life Integration Item-level Results

Work-Life Integration Subscales (Day 8 for Cohort 1 and Cohort 2, RCT Effectiveness)				
	Cohort 1, Day 8 (N=368)	Cohort 2, Day 8 (N=378)	Total (N=746)	p value
**Work without any breaks, Median (IQR)**	1.0 (1.0, 3.0)	2.0 (1.0, 4.0)	2.0 (1.0, 4.0)	0.02[a]
Missing	114	71	185	
Range	(1.0-4.0)	(1.0-4.0)	(1.0-4.0)	
**Skipped a meal, Median (IQR)**	1.0 (1.0, 4.0)	2.0 (1.0, 4.0)	1.0 (1.0, 4.0)	0.08[1]
Missing	109	68	177	
Range	(1.0-4.0)	(1.0-4.0)	(1.0-4.0)	
**Ate a poorly balanced meal, Median (IQR)**	2.0 (1.0, 4.0)	2.0 (2.0, 4.0)	2.0 (1.0, 4.0)	0.07[1]
Missing	109	69	178	
Range	(1.0-4.0)	(1.0-4.0)	(1.0-4.0)	
**Changed personal/family plans because of work, Median (IQR)**	1.0 (1.0, 4.0)	1.5 (1.0, 4.0)	1.0 (1.0, 4.0)	0.03[1]
Missing	111	68	179	
Range	(1.0-4.0)	(1.0-4.0)	(1.0-4.0)	
**Had difficulty sleeping, Median (IQR)**	2.0 (1.0, 4.0)	2.0 (2.0, 4.0)	2.0 (1.0, 4.0)	0.42[1]
Missing	108	67	175	
Range	(1.0-4.0)	(1.0-4.0)	(1.0-4.0)	
**Slept less than 5 hours in a night, Median (IQR)**	1.0 (1.0, 4.0)	2.0 (1.0, 4.0)	2.0 (1.0, 4.0)	0.10[1]
Missing	111	70	181	
Range	(1.0-4.0)	(1.0-4.0)	(1.0-4.0)	
**Arrived home late from work, Median (IQR)**	2.0 (1.0, 4.0)	2.0 (1.0, 4.0)	2.0 (1.0, 4.0)	0.87[1]
Missing	121	78	199	
Range	(1.0-4.0)	(1.0-4.0)	(1.0-4.0)	
**Felt frustrated by technology, Median (IQR)**	2.0 (1.0, 4.0)	2.0 (1.0, 4.0)	2.0 (1.0, 4.0)	0.13[1]
Missing	112	68	180	
Range	(1.0-4.0)	(1.0-4.0)	(1.0-4.0)	
**Number of Hours of Sleep in the last 24 hours, Median (IQR)**	7.0 (6.0, 8.0)	7.0 (6.0, 8.0)	7.0 (6.0, 8.0)	0.19[1]
Missing	109	97	206	
Range	(3.0-10.0)	(3.0-12.0)	(3.0-12.0)	

[a]Wilcoxon

© 2024 Sexton JB et al. *JAMA Network Open.*

eTable 3. Emotional Exhaustion: Linear estimates and 95% confidence intervals for fixed effects when modeling WELL-B intervention on Day 8 (n=421)

Covariates	Estimate (95% CI)	P-value
WELL-B Intervention	-9.55 (-12.48, -6.63)	<0.0001
Baseline Emotional Exhaustion	0.78 (0.71, 0.84)	<0.0001
Sex: Male	-2.77 (-7.90, 2.35)	0.29
Race/Ethnicity (p=0.40)		
White	Reference	
Black	4.39 (-3.35, 12.13)	0.27
Asian	2.90 (-2.98, 8.78)	0.33
Hispanic/Multiracial/Other	-2.11 (-6.84, 2.64)	0.38
Age (p=0.54)		
40-49 years	Reference	
20-29 years	-4.82 (-12.45, 2.82)	0.22
30-39 years	-2.55 (-6.54, 1.44)	0.21
50-59 years	-0.52 (-4.22, 3.18)	0.78
60+ years	-2.58 (-7.48, 2.32)	0.30
Position (p=0.54)		
Others	Reference	
APP	2.79 (-5.35, 10.93)	0.50
Nurse	1.48 (-3.56, 6.52)	0.56
Physician	4.00 (-1.86, 9.85)	0.18
Discipline (p=0.43)		
Nursing	Reference	
Behavioral Health	8.04 (-2.19, 18.27)	0.12
Medicine	1.44 (-4.72, 7.61)	0.65
Others	4.00 (-0.95, 8.94)	0.11
Public Health	2.67 (-5.55, 10.88)	0.52

© 2024 Sexton JB et al. *JAMA Network Open.*

eTable 4. Emotional Thriving: Linear estimates and 95% confidence intervals for fixed effects when modeling WELL-B intervention on Day 8 (n=420)

Covariates	Estimate (95% CI)	P-value
WELL-B Intervention	3.65 (0.99, 6.31)	0.008
Baseline Emotional Thriving	0.79 (0.72, 0.86)	<0.0001
Sex: Male	0.83 (-3.52, 5.18)	0.71
Race/Ethnicity (p=0.25)		
White	Reference	
Black	3.23 (-3.09, 9.55)	0.32
Asian	4.95 (-1.46, 11.36)	0.13
Hispanic/Multiracial/Other	-2.88 (-8.54, 2.78)	0.32
Age (p=0.62)		
40-49 years	Reference	
20-29 years	0.43 (-5.48, 6.34)	0.89
30-39 years	1.59 (-2.01, 5.20)	0.39
50-59 years	2.19 (-1.39, 5.77)	0.23
60+ years	-0.60 (-4.86, 3.66)	0.78
Position (p=0.91)		
Others	Reference	
APP	-1.26 (-7.03, 4.50)	0.67
Nurse	-1.67 (-6.63, 3.30)	0.51
Physician	-1.01 (-6.92, 4.90)	0.74
Discipline (p=0.26)		
Nursing	Reference	
Behavioral Health	-5.70 (-12.99, 1.60)	0.13
Medicine	0.86 (-4.18, 5.90)	0.74
Others	-1.12 (-6.20, 3.97)	0.67
Public Health	-6.30 (-14.28, 1.69)	0.12

© 2024 Sexton JB et al. *JAMA Network Open.*

eTable 5. Emotional Recovery: Linear estimates and 95% confidence intervals for fixed effects when modeling WELL-B intervention on Day 8 (n=420)

Covariates	Estimate (95% CI)	P-value
WELL-B Intervention	3.07 (0.30, 5.84)	0.03
Baseline Emotional Recovery	0.69 (0.62, 0.77)	<0.0001
Sex: Male	2.46 (-2.46, 7.39)	0.33
Race/Ethnicity (p=0.08)		
White	Reference	
Black	4.14 (-0.53, 8.81)	0.08
Asian	5.71 (-0.75, 12.16)	0.08
Hispanic/Multiracial/Other	4.06 (-1.15, 9.27)	0.13
Age (p=0.40)		
40-49 years	Reference	
20-29 years	4.74 (-1.67, 11.14)	0.15
30-39 years	-0.86 (-4.68, 2.95)	0.66
50-59 years	1.95 (-1.46, 5.36)	0.26
60+ years	0.78 (-4.40, 5.96)	0.77
Position (p=0.74)		
Others	Reference	
APP	2.33 (-8.35, 13.01)	0.67
Nurse	-1.17 (-6.37, 4.02)	0.66
Physician	2.16 (-4.57, 8.88)	0.53
Discipline (p=0.33)		
Nursing	Reference	
Behavioral Health	-2.47 (-12.49, 7.55)	0.63
Medicine	-1.90 (-8.06, 4.27)	0.55
Others	-4.11 (-9.21, 0.98)	0.11
Public Health	2.07 (-6.41, 10.56)	0.63

© 2024 Sexton JB et al. *JAMA Network Open.*

eTable 6. Problematic Work-life Integration: Linear estimates and 95% confidence intervals for fixed effects when modeling WELL-B intervention on Day 8 (n=419)

Covariates	Estimate (95% CI)	P-value
WELL-B Intervention	-6.16 (-9.52, -2.79)	0.0003
Baseline Work-life Integration	0.52 (0.42, 0.62)	<0.0001
Sex: Male	-9.49 (-14.95, -4.03)	0.0007
Race/Ethnicity (p=0.06)		
White	Reference	
Black	5.00 (-1.11, 11.12)	0.11
Asian	5.26 (-3.87, 14.39)	0.26
Hispanic/Multiracial/Other	8.82 (1.42, 16.22)	0.02
Age (p=0.43)		
40-49 years	Reference	
20-29 years	-0.38 (-8.65, 7.88)	0.93
30-39 years	-2.38 (-6.91, 2.15)	0.30
50-59 years	2.02 (-2.32, 6.35)	0.36
60+ years	2.25 (-2.84, 7.33)	0.39
Position (p=0.93)		
Others	Reference	
APP	3.70 (-7.82, 15.23)	0.53
Nurse	0.80 (-5.33, 6.94)	0.80
Physician	0.97 (-6.29, 8.24)	0.79
Discipline (p=0.86)		
Nursing	Reference	
Behavioral Health	4.04 (-4.56, 12.63)	0.36
Medicine	0.14 (-7.02, 7.31)	0.97
Others	1.37 (-4.73, 7.46)	0.66
Public Health	-1.54 (-12.48, 9.40)	0.78

© 2024 Sexton JB et al. *JAMA Network Open.*

eTable 7. WELL-B Participant Evaluations

Item	%Agree Strongly	%Agree Slightly	%Neutral	%Disagree Slightly	%Disagree Strongly	n valid
**I liked this session**						
**Gratitude**	84.7	13.3	1.8	.2	0	510
**Work-life Integration**	73.8	23.0	2.6	.2	.4	492
**Self-Compassion**	76.6	20.4	2.4	.6	0	491
**Awe & Wonder**	75.1	18.3	5.4	1.0	.2	482
**Group Level Well-being**	62.1	28.2	8.9	.8	0	472
**The use of interesting/engaging content was appropriate during this session**						
**Gratitude**	81.7	15.6	2.5	.2	0	482
**Work-life Integration**	78.0	19.6	2.4	0	0	464
**Self-Compassion**	80.4	16.6	2.8	.2	0	464
**Awe & Wonder**	79.4	17.1	2.6	.9	0	456
**Group Level Well-being**	74.5	19.9	5.6	0	0	447

© 2024 Sexton JB et al. *JAMA Network Open.*

eTable 8. Participant completion of 5 vs. 4 vs. 3 or fewer sessions of WELL-B

Emotional Exhaustion (by Number of Sessions Completed)					
	five (N=432)	four (N=50)	3 or fewer (N=38)	Total (N=520)	p value
**EE Day 1 (prior to intervention)**	57.3 (25.2)	67.6 (22.3)	60.5 (27.0)	58.5 (25.1)	0.04[1]
N	362	42	24	428	
**EE After 8 Days of intervention**	47.6 (25.0)	54.3 (26.0)	53.2 (21.8)	48.6 (24.9)	0.10[1]
N	430	50	38	518	

[1]ANOVA F-Test

© 2024 Sexton JB et al. *JAMA Network Open.*

eTable 9. Live vs. Recorded Sessions

Emotional Exhaustion (by type of session completed, four or more sessions)

	Mostly Live (N=371)	Mostly Recorded (N=122)	Mixed (N=201)	Total (N=694)	p value
**Emotional Exhaustion (Day 1), Mean (SD)**	56.7 (25.4)	58.1 (24.8)	58.4 (27.3)	57.4 (25.8)	0.76[1]
Missing	39	26	25	90	
**Emotional Exhaustion (Day 8), Mean (SD)**	46.6 (24.7)	49.5 (27.3)	49.3 (27.1)	47.9 (25.9)	0.36[1]
Missing	1	1	0	2	

[1]ANOVA F-Test

Emotional Exhaustion (Day 8 vs. Day 1), Student's t

Type of Session	Estimate (95% CI)	P-value
Mostly Live	-10.8 (-12.6, -9.0)	<.001
Mostly Recorded	-7.2 (-10.7, -3.8)	<.001
Mixed	-8.8 (-11.2, -6.4)	<.001

© 2024 Sexton JB et al. *JAMA Network Open.*

eAppendix. Verbatim comments of RCT participants

Response prompts: What was most helpful or interesting during the series? Any other comments?

A lot of good information and also it had you thinking about different areas of your life

Absolutely wonderful series! It's great to feel like someone cares even if it isn't the people I actually work for. I really needed some positivity in my life right now. Thank you!

Acceptance of feelings. Open honest dialog. Very "real"

Activities - the pace of the material each day was captivating. Not too dull or too hasty.

Activities and all the study information

Actually, many of the definitions like well-being and burnout helped me to understand what I was going through and how to assess myself. All of the recommended practices were helpful too

After all the sessions, I know I need to do some adjusting

Agree with the one comment that it should be mandatory as it is helpful whether you are ready to hear the information or not. It opens your mind and makes you think differently. It hit home more the second time around.

all

All

All

All of it. I feel recharged and am spending more time with my staff

All of it was interesting. It helped me to reset and realize what I need

All of it, honestly! There was more than one takeaway from each lesson. What was really helpful was the quiet time of 9 minutes with the writing. That was incredible.

all of it!

All of the bite-sized improvements we can start making really stuck, the ones that most resonated were expressing genuine gratitude, and putting in an extra 30 min of sleep. And brisk walking for only 15 min, 3 good things. I have really enjoyed the series and have been sharing my experience with friends

all of the evidence provided; tools that are simple and easily used

All of the facts/studies that were done gave insight into why we feel the way we do.

All of the information is wonderful and so helpful.

All of the sessions were helpful. The session on how awe-inspiring moments affect us was especially helpful as are all the tools and resources.

All of the sessions were helpful. The “Awe” session helped me immediately!

All of the tips are so useful. I look forward to seeing the benefits they will have in my life. Thank you!!!

© 2024 Sexton JB et al. *JAMA Network Open.*

All sessions offered useful information and insight. I like the bite-size pieces.

All the different ideas

All the resources. The simple ideas to help improve things.

All the tools! Make myself an “awe” moment every day. Reflect. Positive self-talk, be my own friend.

Amazing webinar series! Well laid out and organized, appreciate the easy access to resources.

Applied to Duke.

Appreciate the encouragement. Can be integrated into a workday.

Appreciated seeing the evidence base that backed up statements. Appreciated starting the session by typing into the chat to get the "wheels turning".

Appreciated the Awe images and thought the telescope images were quite cool to get the point across. But perhaps don't need to use all of the awe images and could even spend time closing eyes and thinking about one of them.

Appreciated the minute to even sit back and think and get into my head vs. the constant feeling of running around with my head cut off / without direction but with some panic. Really thankful for taking the "pause" (even if it was fraught with multitasking and other responsibilities) - but at least to get that minute of headspace to think about things differently, try to reset, try to get a different perspective, try to take a breath. I really appreciated and connected with the need to work on having a more positive mindset, surround yourself with opportunities to not wallow in the blame or frustration, to instead look for gratitude and for awe-inspiring moments.

As research administrators at Duke, we are required to have 12 CE hours per year. I've requested the Office for Education and Research Finance (OREAF) to look at this course and include it in the courses that could count towards our credits. We are a stressed-out group and would benefit greatly from this.

Assessments and the data regarding the topics were very interesting and the simple techniques for adjustment were easy to accomplish. The presenters are sooooo positive!

Awareness and Affirmation.

awe

Awe :).   and it feels wonderful!

Awe & Wonder. Learning to balance work & homelife

Awe and wonder

Awe and wonder

Awe and wonder and gratitude letter

Awe and wonder helped me the most

Awe and Wonder slides, I watched live and again in the recording

Awe and Wonder slides, I watched live and again in the recording

Awe and Wonder was amazing

Awe and wonder was fun to just sit back, relax, and think

Awe and Wonder and Gratitude

© 2024 Sexton JB et al. *JAMA Network Open.*

Awe and wonder.   Now I have to figure out how not to be misunderstood when I feel awe and wonder.   I have been misperceived as talking down to people when I express awe and wonder.

Awe was a blessing and much enjoyed.

Awe, followed by self-compassion.

Awe… it was so incredibly helpful

Awesome topics.

Balance and appreciation

Best emotional wellness training I've had all year!

Being able to get a score back and then hear about tools and techniques

Being able to reflect on myself and my actions.

Being able to reflect on things in the moment

Being able to reflect on things in the moment

Being able to see how burned out I am in a numerical value. It makes you face the truth.

Being able to take time for myself and learn more about self-care and how to deal with burnout.

Being part of something larger…

Beyond excellent! Can't wait for each week's session

Bite-size interventions to use in practice and the science behind them to convince me they work! I really enjoyed this series and plan to do the monthly series as a result. The speakers were excellent and engaging.

Bite-sized exercises and interventions.

Bite-sized tools to use and share with others

Both presenters were excellent speakers and did a very good job talking through the content. The content was relevant and down to earth!

Breaking things down into bite-sized strategies. I especially appreciated the sessions on self-compassion and awe and wonder (the photos were amazing!).

Bryan and Carrie are great presenters/facilitators. Impressive research studies to back the suggested interventions. Now I just need to make sure I practice what I've learned.

CEU's offered for actual nursing use. Great topic!

Commonalities across healthcare, understanding components of self-compassion

Compassion session

Concept of short bite-sized moments of renewal. Loved the reminder about moments of awe. Also, the concept of the social contagion of burnout/well-being.

Concrete doable strategies. Quick lessons that digested a lot of research.

Concrete strategies

Consciously being more grateful for everything and everyone.

© 2024 Sexton JB et al. *JAMA Network Open.*

Cultivating awe and wonder. What a fantastic way to start a day and being able to share an experience can create an engaging conversation.

Data - good to know that there is scientific support

Data on burnout compared to EHR implementations; having to stop and do the exercises; knowledgeable and engaging presenters, bite-sized tangible activities

Definitely looking at the small tools and the length of effects from them

Definition of burnout and ways of reducing it

Description/discussion of the evidence. Very engaging speaker and moderator. Simple exercises.

Different categories (awe, gratitude, etc.)

Different strategies in small sessions. I had not thought of some of the strategies before and like them.

Different tools and strategies to help cope with burnout, self-criticism, and work/life balance which are all significant factors impacting my life right now. I really liked the “little” tips and strategies that were introduced throughout because, to be honest some of the tools are just too lengthy to do during busy days

Doing the activities during the session! Loved the method presented.

Don’t need as much research heavy slides … lots of time spent on studies but a little over the top

Each year is made of days. Each day of hours. Each hour of minutes. And each minute of moments. Purposeful use of those moments brings wellness.

Eager to share with my coworkers and have us do a practice periodically.

Easy to follow

Emotional recovery information. This was a great reboot for me.

emphasis on the minimal amount of time needed to improve so I did not feel like it was another onerous thing to do.

Enjoyed

Enjoyed how I can relate to this in my work and personal life

Enjoyed the discussion and resources helpful

Enjoyed the series. Will encourage my counterpart to attend

entire concept

Everything

Everything actually was amazing!

everything was helpful and interesting, great job!

Everything was helpful and relevant. All the sessions were perfectly organized, and compact but packed with useful data, examples, and actionable tips. If I had to choose the one with the most impact on me personally or was the gratitude session (awe coming in at a very close second)

everything!!!!

Evidence-based and simple approaches to increasing well being

© 2024 Sexton JB et al. *JAMA Network Open.*

Evidence-based suggestions and ideas to integrate immediately into daily practice and workplace

Examples

Examples and pictures

Examples and practice sessions

examples and the science - I can promote something that I know works much more effectively because I believe in it, and that is so important when I'm promoting well-being.

Examples of beautiful places that I had never heard of as a grown person who loves to learn new things, research that provides data about the impact of these interventions

Examples, video, pictures

Excellent presentations! Thank you so much!

Excellent program

Excellent program

Excellent program - I just need to re-review and apply the techniques provided.

Excellent program. What a wonderful gift

Excellent series

Excellent series! So glad I attended

Excellent series. Thanks so much!

Excellent webinars!! Ya'll did an outstanding job!

Excellent, thank you so much!

Excited to be a part of the monthly newsletter.

Exercises and hearing the research to support their use.

Exercises for self-compassion/gratitude. They may seem silly at first, but they were actually really helpful!

Expanding on my knowledge of well-being tools and learning useful strategies that can be used every day.

expressing gratitude helps your overall demeanor and makes you happier

Fantastic series, mental health is so so important and I'm glad I found out about this training.

Feeling gratitude but also sharing with others and how much more impactful that has been when sharing out loud

Finding out how exhausted I am

Finding out how exhausted I am

Finding awe and wonder was a new concept of well-being that I had not heard of before. The examples were truly amazing and did their job of inspiring "awe & wonder"!

Focusing on gratitude and not to overwhelm myself. Tiny bites.

Format was well conceived.

© 2024 Sexton JB et al. *JAMA Network Open.*

Four days in a row during lunch, which is the only break and catch-up time I have, was hard. Maybe spread the info out over two weeks, every other day? Didn’t find the self-compassion session as helpful—a bit too touchy/feely. Would have like more scientific research. Sometimes Dr Sexton talked so fast it was hard to follow, although generally, I appreciated the fast pace

Gaining perspective and having tools to use when stress, burnout, and exhaustion feel like they are peaking.

Getting info about the podcasts was very helpful. Also, I really enjoyed having to stop and think in the middle of the day about someone I was grateful to. It actually prompted me not only to write a letter but to call a friend I had not talked to in a good while. Catching up with her made me very happy. It is just good to stop and be reminded of how blessed we are and to know that we can do it, whatever It is for us but we don’t have to do it alone. We are enough and it's ok to not be perfect but to try to be.

Getting your score- eye-opening

Giving me researched packed reasoning why I need to take better care of myself

Glad I participated!! And not sure if American Psychological Association CEU is available, but if so, that would be my preference. Thank you to all who helped with this valuable program!

Good job guys!

Good mix

Good reminders of simple things I can do that have a meaningful impact on my life. And research to back it up not just personal experience.

good reminders, facts to support the importance of taking them to say thanks, work-life balance, etc. I'm glad I took the 1 hour each day to listen and focus on self-improvement.

Good series. Very informative. Impactful.

Good sessions. Good data. I actually took something from this CE. It was hard to escape mentally while working at a pharmacy input booth to listen to each session.

Good to have recordings.

Grateful for this body of work and research.

grateful to have this content recorded and available to relisten to. Please let us know how we can share the information and support future efforts. I personally believe that every nursing student should have these five lessons and refreshers throughout their training to promote good habits. If you want to consider this partnership with DUSON, please let me know! I'm happy to collaborate with both of you!! THANK YOU

Gratitude

Gratitude

Gratitude and Awe & Wonder sessions were great and just what I needed

Gratitude and awe and wonder

Gratitude and awe I believe I will use most often. I really liked the research provided at the beginning of each section.

Gratitude and awe sessions were the best

Gratitude and self-compassion was most interesting. We often think of others first, it was nice to focus on myself.

Gratitude and the connection with leadership and burnout

© 2024 Sexton JB et al. *JAMA Network Open.*

Gratitude and work-life balance

Gratitude every other month is most effective. Personal debrief at the end of the day. Working together reflection of each other. “The core of mental toughness is actually self-compassion” Brene Brown PhD

Gratitude had the most impact for me. Maybe because it was the first?

Gratitude is always key

Gratitude is so easily accessed.

Gratitude letter

Gratitude Letter

Gratitude tools and awe-inspiring tools were surprisingly effective.

Gratitude was great. The man presenting this information was wonderful. He is so engaging and makes you want to listen carefully.

Great and well -put together resources that should be shared widely for it addresses real-life issues as it stands right now. Thank you!

Great job!

Great presentation! I really enjoyed last week. I was really burnt out and surprisingly in a short few hours, all that changed. I find that the research is really engaging and pulls me into what is going on and then taking action makes a difference.

Great presentations!

Great presenter and moderator. Thank you for offering this wonderful series for free CE credit! Looking forward to the future newsletter and additional resources. I will definitely recommend this to colleagues and family members.

Great program, I wish that I could have viewed all sessions live but my clinical responsibilities interrupted

Great resource. Thank you. I feel it has helped me already.

great series

Great series

Great series

Great series - I enjoyed the inclusion of the research and active exercises to keep participants engaged.

Great series and I have shared with my manager and friends. In two different meetings I have been in, this series was mentioned. Excellent!

Great series that I will be sharing with others.

Great series!

Great series!

Great series!

Great series!!! I hope you offer more.

Great series.

© 2024 Sexton JB et al. *JAMA Network Open.*

Great series. Appreciate having resources available for reference and sharing.

Great series. I would love to get all of my practice employees to complete the series to improve everyone's mental health at my private practice- about 80 employees total.

Great Sessions!

Great sessions! Our team has shared that they really valued having some time each day to focus on themselves and reduce burnout.

Great sessions. I had heard from Mr. Sexton two other times with the leadership series and he is great!

Great sessions. I really appreciated the small bites and the new ways of looking at things.

Great sessions. Thank you!

Great speaker!

Great speakers and content.

Great subject; please add me to additional ones if there are more.

Great training, I really enjoyed it

Great work!

Great work!

Great work/series....

Great. I plan to share with my team.

Group

Group Level

Group Level & Resources

Group resource rounding and the power of positive

hands-on practice using the tools

Having concrete techniques to practice

Having data to back up and support the need to slow down, to pause, to stop trying to multitask every single day. Permission to say "no" because it's better for me and everyone else when I do.

Having time to reflect and do the activities during the presentation.

Having to miss lunch 4 days in a row made it difficult to experience the sessions optimally. I missed two of the sessions because I got pulled into work activities during them. I recommend spacing the sessions out over several weeks instead or starting 10 minutes after 12 so that people have time to grab lunch

Having us do the interventions.

Hearing about the research and doing a self-assessment. I loved all the practical tips and the Q&A. Mostly- I did not feel overwhelmed by the time commitment and each session made me want the next.

Hearing about tools that will enhance my coping skills. I would like to learn tools to deal with difficult people.

Highlighting the power of positive emotions and the concept of social contagion

© 2024 Sexton JB et al. *JAMA Network Open.*

Honestly just being forced to take the time to focus on myself.

Honestly, I think taking the time to consciously think about these topics helped me remember to step back from stressors, see the big picture, and accept that I can and should work on myself and my needs.

Hope to attend more sessions in the future.

How appreciation and awe can have long-term effects on emotions

How gratitude affects my emotions in a positive way

How small bits of time doing these things can have such a large effect

How to obtain self-compassion and Awe & Wonder.

How well done the structure of the series was: the examples, research, and practical exercises.

How you only need to invest in small actions to help you rebalance towards self-care. These small investments have long-lasting results

Huge thanks to you. It might be what keeps me in my job as a hospital Chaplain.

I actually felt like all of the topics were useful and very interesting. they also are very timely. I started an Awe and Wonder board in my department

I am afraid my scores are probably a bit more down than would typically be as a family member got a serious diagnosis last week, and I am pretty worried. Will plan to do a monthly series as I do believe this will be very beneficial to my overall well-being.

I am amazed and grateful that the webinar platform worked so well. I had attended a live version years ago in Boston at ASHP and I loved it. I am grateful there is this version to be more accessible

I am in awe at how much I allow outside influences to influence my mood and behaviors (like skipping breaks and lunch at work). I love how awe has an effect on a person; I didn't realize this.

I appreciate learning the science and seeing the evidence for this approach to well-being

I appreciate that the session was offered during the lunch hour.

I appreciate the additional resources/tools and am looking forward to signing up for the mini studies. I thought the content of these presentations was excellent and both presenters were very skilled. They kept me interested and wanting to tune into the next session - AND I refrained from multi-tasking for a large portion of it which takes a lot these days. Great job all around!

I appreciate the evidence and real examples you are seeing in the data. It is validating and makes me really want to implement the practices we learned.

I appreciate the small, bite-size practices because I do agree that taking something larger than that at this time is unmanageable.

I appreciated being able to watch the recordings on my own time versus having to be present live.

I appreciated that leadership encouraged us to take this series, but honestly, I felt like I had to push myself to not multitask during the sessions.

I appreciated the exercises to try throughout the presentation. I liked the check-ins as it made me feel like I was being held accountable. I really appreciated that these were recorded, I struggled to watch them live with my schedule. There was a good balance of evidence with resources.

© 2024 Sexton JB et al. *JAMA Network Open.*

I appreciated the links to the research and evidence. It helps to validate the importance of this material, especially when we are constantly battling with the C-suite to endorse a work-life balanced culture.

I appreciated the simple tools, breakdown, and links to the evidence and REALLY loved the awe session. Very happy to have found this series and grateful to Duke for offering it

I appreciated the tests to see where I am at and the ability to compare to other healthcare providers' scores.

I believe the gratitude session was the most helpful. Writing the letter was great.

I continue to integrate these practices in my life and the refresher courses help me continue on my well-being journey.

I could listen to both of our presenters talk all day. I need my own personal Bryan to follow me around wherever I go.

I could tell I felt better after watching the episodes and participating in the activities with each session.

I did not like the focus on meditation and the attempt at guided meditation. For me the subject of mindfulness is controversial.

I didn't realize gratitude was a feeling. Sometimes it's good to stop and think of these things and analyze them in my life. I practice some of them but don't really think about it day to day. It's good to check in with myself and realize I'm doing better than I think I am.

I enjoy the focus as a mix of theory, exercises, and fun and interesting examples.

I enjoyed all the tips and tricks to become and remain grateful even in difficult circumstances.

I enjoyed each session. I liked writing the letter to someone you were grateful for who had impacted your life. The time during each session that you had time to reflect was very helpful.

I enjoyed hearing the facts about burnout within this career. I also appreciate learning new methods of managing stress and taking care of myself.

I enjoyed it

I enjoyed learning about the data to support wellness initiatives.

I enjoyed seeing the data.

I enjoyed the awe and gratitude sessions.

I enjoyed the content, hoping g to put the advice to good use

I enjoyed the different aspects of well-being and how we as individuals integrate or lack thereof them into our daily personal and work lives. Stressing the importance of balance

I enjoyed the self-compassion session the most. I found it actionable and very impactful for reframing some of my more unkind thoughts towards myself. I thought writing a letter to myself would feel silly, but it was actually quite cathartic.

I enjoyed the sessions were interactive. I felt examples were relevant and practical. Most helpful my practice or suggestions I could be made and passed on to other individuals or groups.

I enjoyed the short sessions and the recordings in case you want to watch again. Sometimes I felt all the data was overwhelming

I enjoyed the virtual design of how the class was conducted. I'm glad it expanded on resilience and focused on emotional exhaustion, and work/life balance and provided exercises that can make a quick impact.

© 2024 Sexton JB et al. *JAMA Network Open.*

I enjoyed the whole session. It really made me rethink my work habits

I enjoyed the work-life balance the most. The emphasis on the importance of taking even 15 minutes per day for yourself. A walk, meditation, etc. The series reminded me that sometimes you become MORE productive when you take some time to step away.

I enjoyed thinking about how to make the workplace itself more pos for everyone as opposed to just focusing on myself.

I enjoyed this series from a personal perspective, however, plan to share it with shared governance councils so we can reach more healthcare workers, esp. nurses! Thank you for sharing this beautiful work with us!

I enjoyed this series so much! Thank you!

I enjoyed the work-life balance! I gained a lot of weight during Covid. Mostly stress eating! Am now back to putting myself first

I especially liked the mindset of gratitude!

I feel like healthcare has changed over all and accepting these monstrous changes are what today's seasoned healthcare workers struggle with. We know the old system works, which makes it a challenge to adapt to a new system. Thank you for these sessions, as they help refocus our ultimate intentions of caring.

I feel like I could have used some interactive "practicals" - more help with how to put things into practice. the big gratitude letter is great but you can't do that daily. how to implement more bite-sized gratitude into daily life.

I feel like the session I remember most was awe and wonderful and I find myself trying to incorporate that more in every day. I also remember the gratitude exercise, I think I felt the most immediate positive response to that activity. I remember self-compassion mainly because I've reviewed Kristen Neff's work previously. I honestly don't remember much from the work-life balance presentation, maybe I was checked out that day?

I feel renewed after this past week and would like to share this with others.

I feel that we were given almost too much time to complete the activities in each session

I felt the session on self-compassion was the most relatable and likely to have the largest impact for me.

I find myself smiling for no reason partway through most sessions. I look forward to helping others I can now recognize as needing well-B tools.

I found all of the sessions interesting and helpful. Being reminded about the importance of being grateful and compassionate towards myself probably has the most impact on me.

I found all of the sessions to be very helpful. I really liked the self-assessment tool, it provided a lot of information about myself.

I found Bryan Sexton's presentations to be the most engaging and easy to be attentive to. He really has a way of presenting knowledge in a way that is fun and interactive.

I found the segment on gratitude to be very interesting and have implemented it into my daily life. It truly makes me feel better to express my gratitude to people.

I found the self-compassion series to be the most eye-opening in terms of areas I can improve on. I purchased one of the recommended workbooks and am looking forward to trying to improve my thought processes in this area. I found the gratitude presentation to be a good reminder of how this benefits not only yourself but others you work with. I have recommended this course to others.

© 2024 Sexton JB et al. *JAMA Network Open.*

I found the self-compassion session to be the most helpful. I think that's where a significant amount of my burnout comes from. I feel like I'm just terrible at everything I do and it doesn't help my satisfaction with my job. I didn't realize how much that was affecting my burnout until then.

I greatly appreciated this course and would love to learn more about how institutions can ingrain a culture of self-care for staff.

I greatly respect and enjoy both the presenters, their expertise, and the format they present this impactful information.

I had to listen to this during my lunch. I wasn't able to disconnect in the way that allowed for the full experience of the course. It also wasn't in line with my work-life balance and I was usually scarfing down a meal right before I started back to clinic. I will use this going forward, but the course itself wasn't timed right for me, personally.

I have enjoyed sharing the bite-sized strategies with co-workers, family, and friends. The more positivity that you surround yourself with, the better you are guaranteed to feel!! Thank you so much for sharing these concepts with such a huge audience...simply invaluable!!

I have heard these things before but to get in one place is helpful and also, I feel I need to hear these messages over and over to remember to use them. It's never a one-and-done thing.

I have recommended this to several of my burnt-out coworkers. I love it and needed it!

I hope I can make time to watch the other three sessions soon!

I hope to continue this series.

I hope to share a few ideas from each of these sessions with my coworkers on the inpatient unit.

I just really enjoyed all of the information. It was nice to see different topics covered that I had not really thought much about before, such as the session on awe. It inspired me to create a separate Instagram account just for awe that I used to view awe-inspiring things.

I knew some about gratitude balance and self-esteem. Generating awe was completely new to me and really resonated with me.

I learned more tools to avoid burnout.

I like how tangible the advice is.

I like how the Live Sessions were set up. I enjoyed seeing the evidence 1st and then participating in the activity.

I like that we are talking about this stuff and trying to find ways to manage in today's world.

I like the format. Consistency helps with learning and I love the way they teach interesting facts like water bears and goats in trees and tie it back to burnout

I like these sessions!

I like when studies show how concepts work

I liked getting the recordings and doing the activities in the sessions

I liked hearing about the statistics and science behind these sessions.

I liked it all and can benefit from the material.

I liked that all the activities were completed during the sessions and I didn't leave with homework. I also like that the sessions were recorded, and I could go back and listen to them if I needed.

© 2024 Sexton JB et al. *JAMA Network Open.*

I liked that there is scientific evidence to support the use of these resources/well-being practices - and I liked the emphasis that was placed on the positive impact of leaders who are informed about/intentionally offer opportunities to assess (and address) well-being in the workplace - positive impact on staff satisfaction, retention and operational metrics. the bite-sized approach was valuable - made self-care activities seem very manageable. highly recommend to all who are in a leadership role.

I liked that there was dedicated time to participate in some of the tools. Very engaging speakers, never lost my attention!

I liked the act that the statements were backed by science and research and not just "feel-good fluff." As a pharmacist, I read studies to validate statements and rarely do I see the science behind emotional well-being like I did in this series.

I liked the data that was presented that linked the session topics together.

I liked the focus on "bite-sized" techniques and the self-assessments.

I liked the format with both solid evidence reviews as well as time to try practical tools

I liked the gratitude and self-compassion sessions the most.

I liked the gratitude session and the awe and wonder sessions the best made me think of things in a different way. The Gratitude and how it relates to a person that I am grateful for.

I liked the info first activity second model. I do like the hands on and appreciate the engaging speakers

I liked the practice activities in the gratitude and awe sessions the most.

I liked the RAFT format for presenting the topics.

I liked the sessions recorded and the exercises to go back too. Very well done. The resources in the video content for follow up and sharing with others is excellent.

I liked the work-life balance the most as I feel it is easily over looked

I look forward to reviewing all the materials at a slower pace - thank you for sharing them!

I love that these meetings were all recorded so that I could go back and watch without too many distractions. The bite sized tools have been helpful and I hope to use them going forward to keep up my ability to do stuff :-)

I love the perspective of “awe”. Learning to see Awe is something I can do every day.

I love the support and resources. But I have to remember to do the things I’ve learned.

I love, love, love the Raft format. It lowers the BS meter (you know, the unverifiable "expert" that says you need to do this or that), and raises the interest and desire to take action.

I loved all the sessions and feel like I can incorporate the content in my daily life. Especially the Gratitude and Awe & Wonder topics. Hope to see some positive effects from that. Habits take time to establish and benefit from, so I want to take steps to keep the tenets fresh on my mind.

I loved focusing on well-being!

I loved having the scientific evidence presented. Also, I appreciate it being realistic in expectations and not too fluffy. The exercises were valuable without being overwhelming

I LOVED participating in this. I was not able to match up the time of the LIVE course with my lunchtime, so I watched it every night after work. THANK YOU. Please keep making these amazing resources available for everyone.

I loved the awe and gratitude portions of the series best. They helped redirect my perspective on myself and that I need to remember others and the world around me. And the gifts God has given me.

I loved the awe and wonder series. This one in particular I've made a point to find something beautiful in every day no matter how bland or bad

I loved the Awe and Wonder Session.

I loved the awe series. It renewed my interest in unplugging and getting out in nature for the sake of nature

I loved the correlation of work life balance and patient quality indicators. Also, nice tips on how to approach during rounding on employees

I loved the evidence-based approach - it made me more engaged and makes it "believable" from a natural skeptic (though I'm a positive person, I'm still a skeptic!). I have a different perspective on several of the things and it's already been helpful. I can't wait to share this with others and see the effects grow! Thank you, thank you for this awesome program!

I loved the gratitude and self-compassion activities, and I appreciated all of the evidence provided in support of each of these wellness activities. I also liked the modeling of how to implement some of these activities as a leader (for example putting a prompt for the chat that relates to the wellness activity of the day-I am going to do that at my next meeting!).

I loved the gratitude and self-compassion sessions and have already started to implement them in my life. I also discussed what I learned with my adult children.

I loved the gratitude one, that one seems the most "within reach"

I loved the gratitude tools. I also loved the awe & wonder segment.

I loved the interactive Q&A to see other peers experiences

I loved the letter of gratitude the best and absolutely will continue this practice in my life.

I loved the messages in these. It's helpful when they are shorter, but the tools and messages are so beneficial. Thank you for putting this series together.

I loved the session on awe and am inspired to look for it!

I loved the times of self-reflection. Especially writing a letter to thank someone who has made a difference in our lives and describing a time where you had awe and wonder.

I loved this course and plan to participate in the monthly sessions. I'd love to do a mini-fellowship in this program, similar to a 4-week training in behavioral sleep medicine at Duke in 2008 (sponsored by the American Academy of Sleep Medicine). Is this possible, perhaps as a virtual learning experience? Thank you again for everything... my best wishes to you all! Warmly, pat bach pbachpsyd@gmail.com

I loved this. Thank you so much. All healthcare workers need to watch these sessions.

I loved understanding the science behind why the techniques worked.

I missed quite a few, but am looking forward to going back as I am finding this information very informative and helpful

I most enjoyed the gratitude and compassion sessions. All the content was amazing and helpful.

I most enjoyed the gratitude and compassion sessions. All the content was amazing and helpful.

© 2024 Sexton JB et al. *JAMA Network Open.*

I participated in similar training about 4 years ago when I first came to Duke. I was at a different place (better) than now. I enjoyed the content then, and not much has changed, but needed it more now. I honestly felt slightly better these last few days, and attribute that to these sessions. Thanks for the reminder.

I participated in the 2nd day before watching the first day recording (Federal Holiday and I forgot to log into my computer at home). I immediately was telling my co-workers that they needed to watch this series. I am a "data" person and seeing the studies and journal publications. I was thrilled. I am very excited to bring some of the data presented to my facility's leadership to help support an innovation program I am working on. I am hopeful that the hard quantitative data from the presentation/journals will help gain support for employee well-being.

I particularly enjoyed the awe and wonder session-found it helpful.

I particularly liked the last session: awe and wonder. It got me excited again and provided me with a calmness.

I realize the importance of addressing burnout and fatigue head on like this. It does help. Our profession is exciting and dynamic, but is never finished or complete...there is always the ability to pour more hours into it. Thank you!

I realized the great rewards of expressing gratitude. I learned the importance of self-compassion. I appreciated the value of recognizing awe & wonder.

I really appreciated the Awe session as I had not thought about the need to experience awe to rejuvenate myself and take care of my wellbeing. I also liked the encouragement of discussing self-care in front of others to normalize self-care practices.

I really appreciated the information in work-life balance and Self Compassion. The presenters were engaging and interesting.

I really appreciated the self-reflection.

I really connected with the awe session. I love the RAFT model for presenting- it’s perfect!

I really enjoy the way the instructor brings in relatable stories and examples from daily life into the workshop to clearly illustrate concepts being taught.

I really enjoy these sessions. I have brought it up to coworkers and my team is actually looking into doing the monthly session series and that we can then discuss in our team meetings. Thank you to the entire team for these interesting, educational, and fun sessions.

I really enjoyed all discussions but really related to the moments of Awe. I found this to be inspiring and true. I have taken this to my staff huddles to share.

I really enjoyed finding out the stats on work/ life balance and how it can seriously affect patient care.

I really enjoyed the awe and wonder. It reminded me to look for the good that is around me instead of just seeing the bad. Moments don't need to be huge just recognized.

I really enjoyed the Compassion and Awe and Wonder series. I felt those helped me the most and the activities were wonderful.

I really enjoyed the entire series. It was nice to take a few minutes to get your mind off work and reset yourself. It was very relaxing and eye opening.

I really enjoyed the more recent data updates. Some really interesting findings!

I really enjoyed the RAFT layout.

I really enjoyed the session on Awe and realized how I have already been utilizing awe. I want to increase this now that I am aware that it has a real quantifiable impact on my wellbeing!

© 2024 Sexton JB et al. *JAMA Network Open.*

I really enjoyed the sessions about gratitude and the science of awe (this was my favorite). I think all the sessions are a good reminder of how to reconnect with yourself and the big picture of life.

I really enjoyed this series it helped me understand how all areas work together to help me thrive at my job and how to have work life balance.

I really enjoyed this series. I am not a "touch-feely" individual, I set high standards for myself and do not have much self-compassion. I really benefitted from this more than I thought I would.

I really found John to be enthusiastic and engaging with all the material he presented. I would listen to his seminars every day…okay maybe more like once a week at least :)

I really got something out of the Awesome and Wonder, and the Self compassion modules. I am looking forward to utilizing what I have learned in both of these sessions Thank you so much for these presentations!

I really like all of the slides in Awe & Wonder

I really liked pausing to do the reflection pieces. I also really loved all the pictures of the macro and micro sources of awe. It was AWESOME!

I really liked reframing gratitude as forcing us to acknowledge the positive and as a gateway to hope...really useful. Also situating it socially. Honestly, I had gotten tired of gratitude journals and this session really breathed new life into it.

I really liked the “Awe” session! Also, I liked the accountability aspect of the quizzes. It really got my attention. I liked the bite-sized aspect as well (baby steps)…get the bike out of the garage!

I really liked the activity on writing the gratitude letter.   I’m searching for the right cards that I want to use to send. Loved the awe session. I’ve created in my mind my “place” to hopefully ground me when stressed. Wonderful tips to use in my future. Thank you for offering these sessions.

I really liked the Awe and Wonder, I learned new things in this session and it was my favorite of the series but they were all great. It was great to hear the research

I really liked the awe session. So many times, I feel like I’m wasting time when I look to nature and try to notice small, marvelous things. It makes me feel good and now I know the science behind it and I have been doing it without realizing it.

I really liked the gratitude session!

I really liked the self-compassion talk. I am my harshest critic, and it was helpful to learn to be as gentle with myself as I am with my friends.

I really liked the times where we could reflect on our own experiences and write things out. My favorite activity was when we wrote a letter to someone who greatly impacted us.

I really liked this series and I am excited to use these tools with our pharmacy staff. Thank you for sharing!! :)

I really think I benefitted from the awe session

I remain interested in following along with the wellbeing webinars as they come along. Thank you!

I think it is always good to hear about these techniques and put them all together as an action plan. Every option does not work all of the time but we have an arsenal of ways that we can address burnout. I thought the research was interesting and gives credit to the fact that these techniques work.

I think just hearing the information and knowing so many others are in the same boat. Small things that I can do each day to help keep my mental state healthy is a huge win!

© 2024 Sexton JB et al. *JAMA Network Open.*

I think that the sessions were awesome and will definitely share them!

I think the Gratitude and Self Compassion session were helpful.

I think the showing gratitude was helpful. I sometimes forget the people that are there for me when I'm feeling very stressed.

I think writing the letter to someone I feel grateful for was really helpful. I shared this letter with my person and she was very touched and appreciative. I also loved the writing a nice/forgiving letter to myself was helpful and moving as well.

I thoroughly enjoyed the built-in activities that allows you to reflect. I have. Even trying use these skills already. Dr. Sexton is also an amazing presenter.

I thoroughly enjoyed the use of images and humor during the series.

I thought self-care was mani/pedis, massages, reading a book, etc. And while they can be, they often feel like one more thing I have to schedule, sometimes don't get scheduled for that very reason and if they do, don't have a lasting positive effect. The tips in this are so bite sized and don't even need scheduling. They can be slipped in seamlessly to my day and I think have more lasting power than the mani-pedis and less expensive!

I thought the self-compassion was the most helpful for me personally

I thought this series was outstanding! Some of my work involves wellness and work/life balance at the VA so I am used to hearing about some of these topics. However, the way it was presented was so fresh, and interesting, and new. I really enjoyed the research portion of it and also the little activities that were thrown in each day.

I took away the ability to choose to pause and reflect or meditate to stop the negative effects of work stress.

I took the time to listen to these sessions & focused on how to manage my workload.

I want to share with all health care workers.

I was a first assist surg tech for 20 years and burned out. Taught many interns, assisted fellowships, been through hospital acquisitions, and seen a few surgeons pass away that were dear friends. Had something been around like this I may have dug a little deeper and persevered. Now an analyst and really like what I do on the flip side. Thank you Bryan and Carrie!

I was hoping it would give me options that I could influence my own well-being and did not depend on someone else. this offered me these options.

I will continue to follow the site for future sessions and tools. This was very helpful and re-energized me in my job.

I will definitely be doing these again!

I will definitely put the resources to good use. I plan to rewatch the series at least quarterly.

I will recommend this to peers

I will try to implement in my department

I wish more people could see this series because I think it would truly make a difference

I work in a doctor’s office setting so it was fairly easy for me to take my lunch break during these sessions. But for other healthcare workers in a hospital setting I think it would be hard for to do the sessions during the lunch time. 8pm/8am sessions might work better for those that work in hospital. Or shorter 30-minute sessions?

I would have liked to have a bit more on how to engage on this topic with family, friends, and co-workers who are resistant or insist they don't have time.

© 2024 Sexton JB et al. *JAMA Network Open.*

I would like to learn skills to deal with difficult people in the moment without reacting to triggers.

I would love to hear you talk about empathy and communication and presence and how we can be empathetic and good listeners without becoming fatigued.

I'm excited to share this with others - I love the science and the engaging way this program is delivered. This will no doubt have a meaningful impact to anyone who attends!

I'm trying out the podcast 10% happier and I'm thinking about self-compassion and how that is tying into my level of burnout.

I'm looking forward to the monthly news letter

I've had lots of experience with coaching and this just further solidifies concepts I've learned. The shift to positive emotion is available to us more than we know and I look forward to practicing it even more. Thank you for all of your hard work.

Importance of gratitude

Incredible program - needed by so many.

Insight timer app is great. Thanks for suggesting. Awe session was great. I want to learn more and will do more reading. Thank you for providing additional resources.

Inspirational. I enjoyed the tool s given along with the evidence base. I have shared the recorded sessions with family.

Intentional protected time to engage in the exercises.

interactive exercises backed up by research

Interesting that leadership really does have such a profound impact on psychological wellbeing.

It felt nice to take the time for myself over lunch to focus on me.

It got a little tiresome to do the emotional exhaustion and work-life balance scales with each activity. There was very little change from day-to-day. Could they be done at the beginning and end of the series rather than each session?

It is wonderful that you are able to provide these sessions for those of us outside your organization.

It made me check in with myself

It was a good series. Work unfortunately prevented me from participating a few days that I find humorous as this was the reason for doing the sessions. Life is very unpredictable, and work is a mandatory part of life. Just need to keep them balances for a good healthy life.

it was a good way for me to reflect on the decisions I make with sleep and diet, it reminded me that I need to take care of myself and not always poor all of myself into work.

It was a great reminder to pay attention to self-care and work balance

It was all great. I like the RAFT format. Very helpful. I really enjoyed the session on self-compassion. And the information about will power peeked my curiosity, too. And the distinction between recovery and thriving so helpful.

It was all very helpful with good reminders and tips that are relatively easy/quick that can affect change.

© 2024 Sexton JB et al. *JAMA Network Open.*

it was difficult to view this series during my lunch hour. I'm trying to make lunch and eat lunch while paying attention to this. And I really wanted to pay full attention to this but I couldn't because of all the things going on at lunch.

It was helpful to be able to quickly measure my own well-being in regard to balance

It was helpful to hear an external individual acknowledge the last few years and the difficulties it has brought. That alone was helpful. The nice part however is the ability to use these resources in an ongoing format to optimize things.

it's interesting that learning what I need as an executive leader and realizing what those who work with me need sometimes can be a work. so, I am trying to balance the two. let me gratitude giving that fills me go toward a staff person

Just being made aware of the enormity of the problem among Healthcare workers. Couldn't even concentrate fully because of interruptions during the sessions. Will enjoy reviewing them again.

Just doing multiple choice questions

Just good validation about how feeling. Also, loved the options to work through/cope

Just hearing out loud that everyone is having trouble these days and there are ways we can help ourselves

Just learning about different areas of wellness.

Just to say thank you! This has been so insightful and helpful! Looking forward to more!

Keep it up- thanks!

keep them coming! love the cadence and great topics!

Keep up the good work.

Knowing I am not alone

Knowing that I am not the only one that may struggle at times. Right now, things are good.

Knowing that I’m not alone.

Knowledge and sharing ideas - different ways of thinking

Last week was world mental health day--- at a time where healthcare worker mental health awareness is at its peak in popularity, the health system that I work at sent an email out to donate to the new mental health hospital that recently opened. This is the type of culture that healthcare businesses need to change! When I opened the email, I was in shock. What could have been a sincere message to the employees, at all levels, that our health system values its employees and takes their mental health and wellbeing seriously, was clouded by asking an already drained population for money. It was embarrassing. How do we make a change to balance all sides of the pendulum???

learned so much about caring for myself. Thank you for this series.

Learning about how self-compassion can help prevent burnout

Learning about self-compassion. Interactive QA sessions.

Learning about the importance of awe and wonder.

Learning about work life balance

Learning how impactful a brief intervention (like writing a letter of gratitude) can be. That is incredibly encouraging to me in the work I do to support the wellbeing of health care employees.

© 2024 Sexton JB et al. *JAMA Network Open.*

Learning how to balance work and home

Learning how to understand the exhaustion and deploy tools to help combat the negative emotions and also that it's ok to not be perfect and also to say no for well-being reasons.

Learning introspection (to pause & reflect) is probably the most overall helpful tool

learning that it is ok to take time for myself and put myself and my health and needs above some stressors at work. Also learning how to disengage and look at the personal relationship or interactions as part of a stressor or stress reliever

Learning the bite size techniques and the research to support them. It was manageable and therefore more likely for me to make and take time for my wellbeing and help my team!

Learning the research was validating and interesting. I enjoyed the hands-on aspects of the sessions and things that I can actually continue to do in my life. I have been more aware of how I can use gratitude and awe to help my outlook.

Learning to have more self-compassion for myself and trying to find alternative resources to feel less burnout

Learning ways to gain balance in work/home life.

Lessons in gratitude, and the work life balance teachings.

Letter writing in gratitude session

Like re-framing wellbeing as capacity for positive experiences rather than being happy. Liked that the interventions are brief and that multiple interventions were provided so attendees have options to choose from.

Liked seeing the research!

Liked the awe series a lot!

Liked the series!

Liked the series!

links and web pages to content

Looking at some of these often-used terms in new ways to make them more palatable and positive. Feeling hope towards a positive environment when systems look at these areas and see them as important.

Looking at things in a different perspective and taking time to be in the moment.

Lots of information with both broad and narrow examples, friendly and energetic leaders, suggestions seem realistic

Love the Awe walks and awe writing

Love the session on awe. Feels like a simple way to bring me back down to reality instead of living in the scary/stress all the time.

Love this series, thank you!

Love this!! Everything about it!!

Loved how calm and relaxed these sessions made you feel.

Loved it

loved it!

© 2024 Sexton JB et al. *JAMA Network Open.*

LOVED it!

Loved it! Thx!! So glad I participated.

Loved RAFT, helped me formulate the story of recovery.

loved the awe & wonder

Loved the awe and wonder. Great lessons all around, even the reminders for what I already know but get lost in the day to day sometimes

Loved the awe class and the practice of 3 things to be grateful for each day. Also loved the water bears!

loved the examples in last session!

Loved the gratitude letter! and so much more. I've participated in various sessions of yours and really enjoyed these 5 intense sessions, great activities, definitely encouraged reflection on improving wellbeing in my own life but also that of co-workers and others at work and of family! Love the awe session - reminds me I can get so busy that I don't go outside enough. THANKS so very much for providing this to us!!! Awesome work and love the evidence.

Loved the idea that the opposite of depression is not happiness, but hope. Trying to imbed self-care and gratitude into my daily routine. Awe is important and I've seen other research on it, specifically Dacher Keltner. I think more could have been done with this module.

Loved the pace...quick bites of very beneficial info...quick and easy to absorb

Loved the series and am sharing with all my healthcare friends

Loved the session about awe! The pictures and how the sense of awe can increase our well-being was great!

Loved the sessions. I hope you can reach multiple health care workers in the nation.

Loved the stories and the facts. Very easy to use all these things in my daily life.

Loved these sessions, so glad that I heard about them and was able to attend each session live. Thank you for this valuable information!

Loved validation about stressful work place and how it impacts me

Loved what I saw so far, too busy to attend all, but glad to have recordings.

Loved your program. Bought the gratitude journal and can't wait to use it

Man. So much. I have heard some of it before but renewing my self-compassion displaying gratitude more and finding awe in nature

Many of the theories and concepts presented in the series are not earth shattering, and for the most part, commonly known. However, these presentations framed these within the context of evidence and research, which is very helpful and eye opening.

Many of these things I had heard before or suspected where true, however, learning the research that backed it up was very interesting.

Matching the research to the tools and activities and doing the activities right in the meeting.

Modeling gratitude in front of my kids

Moments of awe & wonder was my favorite. Even in the 8 day follow up, I loved the analogy of the starfish comparison of how we can clean up debris in our own life.

© 2024 Sexton JB et al. *JAMA Network Open.*

Most helpful was being able to watch from a recording or revisit the recording. Thank you!! Most interesting was learning out burned out I am. The awe session is wonderful and can make a difference quickly in a person. Thank you!

Most helpful was the exercise of writing the thank-you letter on day 1

Most helpful was the way it was all presented. Easy to listen to, easy to follow, etc. Self-compassion and gratitude were both awesome sessions. I did like the hour each day vs cramming all sections into 4-5 hours. Easy to listen to an hour each day. I really enjoyed these presentations.

Most helpful: bite-size tips on how to achieve resilience/well-being; Interesting: the 'awe' session with its pictures.

Mr. Sexton is very informative and easy to watch. His excitement about the topic radiates off the screen.

My favorite session is the "Awe & Wonder".

My favorite was self-compassion, I found the exercise to be inspirational. I didn't really know what self-compassion was, until I experienced this exercise. I would say life altering in a good way!

N/A

N/A

N/A

N/A

N/A

N/A

N/A

Na

Na

Na

Na

Na

NA

NA

New content on awe

No

No

No

No

No

© 2024 Sexton JB et al. *JAMA Network Open.*

No doubt the gratitude letter. It gave me a chance to acknowledge and say things that otherwise may have gone unsaid. I also loved the recorded session on self-compassion. Hat speaker was wonderful. And Brian was so charismatic and easy to listen to.

none

none

None

None

None

None yet

Not at this time

Not at this time. Really enjoying the series!

Nothing at this time

One information shared about self-compassion

openness and candidness

Other resources identified.

Overall techniques and awareness.

Overall the information I gained and the resources backing the data were great to see. I appreciate the individuals who took the time to offer these as burnout and exhaustion are huge in healthcare right now for all disciplines. I have passed along recordings to my leader. Thank you!

Overall, a wonderful series of presentations. I enjoyed my time learning and to show the EBP was very helpful to present the "why" behind the information.

Overall, all the sessions were very interesting and useful. Love the studies and research and findings. Spouse and your colleague, local leaders have lots of contributions.

Pausing and taking the time during the session. I recently quit my job primarily due to burnout and I have been trying to do this work. Sometimes it's also appreciating that your work environment won't change and if you can't be well and be successful then you need to move on

Personally, I enjoyed the opportunity to write a gratitude letter to a coworker. I encouraged me to get my thoughts down on paper and then share them with my colleague.

Perspectivism, the last class really drove that point home for me.

pictures and charts/graphs

Please continue this series/format and offer it frequently. It's perfect for busy physicians, who need it desperately! I would love to see the same for some/all of the other emotions. Thank you for this important content. So well-done.

Please continue to send educational offerings. kbmccormack@carilionclinic.org

Please offer these regularly -- I'd like an annual refresher!

Positive outcomes and encouragement for self-care. Thanks!

Positive Rounding vs Safety Rounds

© 2024 Sexton JB et al. *JAMA Network Open.*

Positive tips to help people find a way out of burnout. Reinforcement about focusing on the good in life and to try to see good in simple moments.

Practical strategies to employ and share with others

Practical tips

Practical tips

Practical tips that are short. eg send a text to someone that's not expecting it and share something positive. a short brisk walk can combat negative mood.

Practical tools that I can use right away and the research that backs up the theory behind them.

Presenting evidence and using quick tools. Amazing, engaging presentations!!

Probably the incorporation of data from the studies. The speakers were great too.

providing a variety of tools from which to engage and adopt in my personal and professional life. gave me ideas on how to tweak to incorporate them - sometimes not exactly as highlighted in the presentation but got me to think about how I might embrace them. speakers are very engaging. loved the research to support everything...this is science and data-driven and backed.

Provision of the science and biology of self-care

Question: is it ok to share slides with family, friends?

Questions answered re male v female statistics. Statistics shared. Interviews shared.

RAFT, I like the breakdown, and the science behind why these activities are factual

Really appreciate this program and highly recommend to others.

Really appreciate your well-being series.

Really enjoyed the series and felt it hit the “reset button” at a good time

Really enjoyed the sessions and have things I have taken away that have had a positive impact on me.

Really enjoyed this series and found it extremely helpful.

Recognizing that I have choices and that I need to prioritize finding the gratitude, compassion, awe and WLB for my own physical and mental health.

Refocus on self-care

Reframing the focus of my attention from me to others and the awesome world

Refreshing just to see this topic addressed and discussed. These sessions felt like an oasis in the middle of the day - and I really appreciated the friendly discussion.

Reinforcement, fact bases processes

Relating the topics to use as individuals and having a survey to evaluate ourselves.

Reliable and dependable start and stop time. Built time into the session to complete reflection. Evidence supporting the strategies is reassuring and motivating.

Remembering that others are struggling with not enough time and that stopping to enjoy the little things is one of the most important things we can do for ourselves. In the Awe and wonder I liked the sunlight coming through the tree's analogy.

© 2024 Sexton JB et al. *JAMA Network Open.*

Remembering to take a moment to care of myself daily.

Reminder to be grateful of the little things and be kind to ourselves.

Reminders about the importance of balance and tools to help find balance.

Reminders to care for myself like I do for my good friends. Reminder to find AWE

Research to back the tools you suggested

resources to re-review later as I have specific areas I need reminders of how to best cope.

Review of evidence from the literature.

scientific evidence behind everything

See all the data that shows it's not just me and then being able to do positive interventions.

Seeing the abundance of evidence. Bite sized interventions.

Seeing the research and then practical applications.

Seeing wellbeing as "the ability to do things" rather than "being happy" as I have seen it in the past. The emotional exhaustion portion resonated with me. I would like to note that my sleep in the last few weeks has been poor due to being a nurse leader leading a team transitioning to a new medical record. We are 13 days away from go live, so sleep and work life balance have been suffering.

Self-assessments and comparisons

Self-care and techniques. "How to ".

Self-compassion

Self-compassion

Self-compassion

Self-compassion

Self-compassion

Self-compassion

Self-compassion

Self-compassion - the thing I struggle with the most.

Self-compassion and awe and wonder. I gained skills I can use and share with people.

self-compassion and awe walks

Self-compassion had concepts that I have t heard before. The exercises were unique and I definitely felt different after each.

Self-compassion is a must. Moments of awe are therapeutic and expressing gratitude is always a good thing.

self-compassion section

self-compassion.

Self-reflection

© 2024 Sexton JB et al. *JAMA Network Open.*

Self-reflection and feeling somewhat euphoric after each session

Self-compassion was most helpful to me as a strategy and I can remind myself to have as much grace with myself as I do with others.

Self-compassion. That was an eye opener

Session on Gratitude

Session on gratitude. I really liked the activity in this session and it really did make me feel better.

Sharing and explaining the research used to support sessions (especially for the work-life balance session). The Q & A I wish was recorded for each session (the self-compassion one I did get to attend live and they were helpful). The awe and wonder samples were awesome. I consider myself well-traveled and did not know of these examples.

Simplicity of the tools that have lasting effects

Slow down, pause reflect and see the awe around you. Allie yourself some grave and don't listen to the negative self-talk. I need to work on getting more sleep

Small focused intentional activities can make noticeable improvements in your wellbeing.

So far, the gratitude and how it has to be done continuously in order to keep the effects of it to work well

So grateful for this opportunity!

Some of the evidence-based studies. How it relates to real life

Some of the slides backed by commentary and positive focus on work life balance. Specifics on how to get there!

Speakers were excellent Data and evidence was relevant and appropriate Activities were really helpful and provided insight

Specific strategies and tips

Steps to unwind and find positive aspects in the workplace

Such a great course

Such a great session!

Such good information!! Very engaging and I appreciate this being offered to us.

Surveys during

Taking a step back to really re focus on wellbeing and making it a priority in my everyday life. The work life balance session was the most rewarding, and having better self-compassion.

taking the surveys for myself

Taking the time to actually practice writing and reflecting.

Taking time out to evaluate myself

Taking time to do the series, most valuable was the gratitude/ and gratitude exercise. I found myself really pumped up for the next in the series, but I wasn't as thrilled.

taking time to focus on me. learning the research and practical tools to help me improve my wellbeing

Talking about different tools to use. How it affects all of the healthcare system.

© 2024 Sexton JB et al. *JAMA Network Open.*

talking about real life examples, activities

Techniques taught to reframe how you think of things in a more positive light

Techniques to address my issues and concerns

Terrific series!

thank you

thank you

thank you

Thank you

Thank you

Thank you

Thank you

THANK YOU - this was helpful to rejuvenate my outlook. I'm working on celebrating the "little wins" throughout the day - rather than waiting on the "big win" that might happen once a month. smaller sustainable "bites" to help improve "what really matters".

Thank you for this.

Thank you for all of the information and the upbeat positive presentations

Thank you for all you did in this amazing series.

Thank you for all you do

Thank you for all your work in this area. It is so critical in these times of unprecedented stress and burnout in HCWS. HCWS need easy, accessible and long-lasting strategies and your research is guiding folks to those exact tools.

Thank you for allowing me to attend

Thank you for developing this series!

Thank you for doing and sharing this meaningful work!

Thank you for doing this and allowing health care workers from all over to experience this! Y'all are gold!

Thank you for doing this and I'm happy I got to be experience some of it. Definitely a mindset shift in the works.

Thank you for doing this. I hope to do this with my team.

Thank you for doing this. I'm a new PA in Duke primary care, but also a 37-year-old woman with a whole crazy life (three kids and marital strife and aging parents and bills etc. etc. etc.) so balancing everything has been somewhat of a struggle and this is the sort of content that grounds me. I normally get it through audiobooks, meditation apps, and podcasts, and I've been so happy to have this content from Duke. Thank you for the work you do, truly.

Thank you for having this program online so anyone can attend from their clinic or other jobs at Duke.

thank you for incorporating the science that supports the struggles we face.

Thank you for making this available to healthcare workers! I needed this time, and I would love to see more programs like this. People in healthcare and the community are terribly burnt-out.

© 2024 Sexton JB et al. *JAMA Network Open.*

Thank you for making this available.

Thank you for making this available. I plan to share it with other healthcare workers.

Thank you for making this content available - and free! This was a refreshing break in my day.

Thank you for offering this free!

thank you for offering this program

Thank you for offering this series.

Thank you for offering this series. I look forward to receiving the recordings.

Thank you for offering this to us!

Thank you for prioritizing physician and healthcare wellness through research!

Thank you for providing the links and slides for the sessions!

Thank you for sharing the literature and giving us a chance to practice these tools

thank you for sharing this I formation.

Thank you for the opportunity and sharing your research with us.

Thank you for the opportunity.

Thank you for the well-presented, organized, and supported meetings.

Thank you for this gift! You are both great speakers!

Thank you for this offering. I found the research and the activities to be very helpful.

Thank you for this opportunity and the resources

Thank you for this series and the enduring materials so I can refresh myself.

Thank you for this series! This is so needed by our society as a whole. We are struggling each in our individual life, and it is good to know ways to increase not only our own well-being, but that of others around us. My family has been instituting some of the suggestions, and it is making a difference in our outlook, our positive sense of self, and our interactions with one another. I cannot thank you enough!

thank you for this series.

Thank you for this series.

Thank you for this series. I will share with my team.

Thank you for this series. It came at both a good and bad time for me as family issues related to an illness have been at an all-time high, with most of the work and stress falling to me. This was not a normal couple of weeks for me and that will be reflected in my responses. This information was helpful and much needed for me.

Thank you for this!!!

Thank you for this. I wish Senior leadership at my organization would take this training

Thank you for what you do. It’s very helpful.

Thank you for your important research and its impact to healthcare personnel at a time of significant need. Well done!

© 2024 Sexton JB et al. *JAMA Network Open.*

Thank you for your research, time, sharing, kindness, and I am grateful for this opportunity.

THANK YOU for your time leading these sessions. I know I am taking away so many things that I will incorporate into my day to day.

Thank you so much for doing this!

Thank you so much for having these sessions, I think there is some really great material I am able to share with my colleagues and add to my own presentations and continued learning.

Thank you SO MUCH for offering these sessions. The research becomes wasted if we don't share the information and resources to the masses, and these are resources that I want to help shout from the rooftops to get to everyone! You all are doing really important and soul-warming work. Thank you, thank you, thank you.

Thank you so much for this and providing us recordings so we can re-watch.

Thank you so much for this course.

Thank you so much for this opportunity.

Thank you so much for this valuable series! Having to take the surveys with each class was a bit laborious for the participants, but I understand why you did it that way. If possible to do one pre-survey at the top of the series and one post-survey at the end I think that would help a lot.

Thank you so much!

Thank you so much. I enjoyed each session immensely & you both were fun to have class with.

Thank you so much. This was great and I know I've learned some tools to do with my own team.

Thank you this is excellent. I am intrigued by the prosocial emotions, particularly awe and its relationship to perceived time.

Thank you! ten!

Thank you, already recommended this course to so many people

Thank you, as always, for bringing light to these topics and helping health care workers understand the impact of our work and how it impacts our daily lives.

Thank you, this information was very helpful.

Thank you, this was helpful and refreshing. I was hoping for more advice on how to improve work-life balance. How to schedule things in or make more space for positive activities/hobbies/breaks.

Thank you! Always looking for things I can take back to my team to help reduce stress/burnout.

thank you!

thank you!

Thank you!

Thank you!

Thank you!

Thank you!

Thank you!

© 2024 Sexton JB et al. *JAMA Network Open.*

Thank you!

Thank you!

Thank you!

Thank you!

Thank YOU!

Thank you! Good stuff!

Thank you! I attended the live in person meeting on April 21 that we had for our research leaders in the department of surgery. I enjoyed your energy that you and Christen gave to the group and these zoom sessions.

Thank you! Much needed right now.   This is the first time I experienced burnout and it is not fun and this is helping me get back to my normal.

Thank you! I really appreciated that this was offered for free. I also appreciated the "bite-sized" approach: so much well-being advice is rooted in things that feel unrealistic to me or things I just don't really feel like doing (even if it was in my best interest): getting a lot of exercise, eating super healthy, etc. This felt more realistic.

thank you! thank you! thank you! this is excellent information and I plan on viewing those sessions I missed this week. Best-

Thank you!! This has been super helpful!

THANK YOU!!!!

Thank you.

Thank you. I must transfer in bite-sized pieces... our staff needs this, but the team members are reluctant. Thank you for evidence-based information.

Thank you. Looking forward to monthly sessions.

Thanks for a great series!

Thanks for an interesting and interactive course.

Thanks for having these sessions!

thanks for providing the tools in an easy to follow format

Thanks for sharing this information. It has been really beneficial and came at a good time

Thanks for the information. I am already incorporating some of it into my daily life and plan to keep the information on the forefront of my mind.

Thanks so much!

Thanks so much! Really enjoyed this training

Thanks so much. Very grateful for all you do, Bryan and Carrie!

Thanks you for doing this research and for recognizing the need for healthcare workers and many other professions.

Thanks!

Thanks!

© 2024 Sexton JB et al. *JAMA Network Open.*

Thanks!   If it worth anything to know, my score didn't dramatically improve based on the set questions asked, but I do feel that it was helpful in improving my overall emotional well-being.

That I was able to commit to engaging in the entire series!

That my well-being is my ability to do stuff. It really opened my eyes to some things outside of work that I am struggling with.

That small changes effect big outcomes.

The "tests" were helpful to kind of put things in perspective on a daily and then weekly basis.

The ability to rest and be passive for an hour during my workday :-)

The act of writing words of appreciation to someone. The video of the participants calling g and telling their person how they appreciated them was stunning. I sobbed.

The activities

The activities

The activities that "forced" us to apply the lessons learned during the sessions. I may intend on doing them later "when I have time" but realistically, that might not actually happen.

The actual takeaway strategies to improve well-being are the most helpful! For example, the "3 positives, 1 negative" approach from today's session. Of course, the science is important and persuasive, but having actual takeaways to work to improve your well-being is what I really held on to for each session.

The approach- bite sized interventions- is very powerful. I don't feel so overwhelmed when I feel stressed- I feel empowered to bounce back. The evidence is there and I think it works

The Awe & Wonder session was absolutely amazing! I also really really appreciate all the evidence-based information that was provided. Also, I appreciate the presenters staying on for extra time to answer all the questions!

The Awe & Wonder was most helpful. Looking forward to implementing this with my team.

The awe and wonder I felt had too many pictures, the point was proven after the 10th photo. I struggle with accepting that self-compassion is okay. I've always lived with no excuses, do better.

The Awe and Wonder was a new concept to me and I had never heard that before.

The awe class was the best for me, it reminded me of why I am alive

The AWE session was my favorite and I used it all this weekend and what a positive powerful experience

The Awe session was very cool!

The bite sized activities to utilize during my team meetings

The bite sized tools

The bite-sized practices and self-evaluation time.

The bite-sized strategies shared.

The concept of bite-size practical tips and how to implement beyond just individual level. Well-being can seem daunting to tackle though this study makes is seem doable. The science behind the practice was also amazing

The connection to the science of how small interventions can have large effects.

© 2024 Sexton JB et al. *JAMA Network Open.*

The content was all very interesting. I appreciated the fact that the content overall gave me time to reflect on my job and how to have a better work life balance, and just be overall happy with life across the board. I also appreciated the activities; especially surrounding gratitude.

The content was excellent and applicable in all of the sessions. I found the self-compassion most helpful.

The contents are well organized to present interesting and convincing concepts

The daily self-assessments.

The data about impact of a poor work -life balance. The impact gratitude can have.

The data speaks to me, I like having information, maybe to rationalize my self-care

The data that was shared and the way it was delivered. I appreciated the time to apply the information during the presentation.

The data to support the information was so impactful.

the different activities to really focus on the subject at hand and put it to use during the presentation...not like so many that suggest you do it later on. Well done!

The different aspects of each session but the importance of each of them.

The discussion about self-compassion

The education you provide normalizes me and helps me understand my own feelings and frustrations.

The emphasis on finding ways to focus on self-care

The engagement

The entire program. Love the RAFT framework, graphics, presentation style, interactive component, and Q and A. You were able to pack so much into a 60-minute session... it was amazing! Your personalities and personal reflections also made an enormous difference as presenters.

The entire series gave my food for thought of how best to handle work-life balance to prioritize what’s important and to prevent burnout.

The evidence and resources and recordings.

The evidence base of each session was helpful to know that even small changes to improve your health/wellness can be effective. I thought some of the facts included in the awe/wonder session were very interesting and really kept me entertained throughout the session.

The evidence behind the exercises that show effectiveness and a chance to do the activities.

the evidence you provided with each topic and the self-reflecting tools done during each session

The evidence-based approach

The examples and the letter of gratitude

the examples with references

The excitement of the speakers when presenting materials, use of universal analogies and relative disclosure, did help be recall and reflect on my own day to day life.

The exercise on self-kindness... Yeah, I was crying and totally appreciated the new perspective

The exercise on writing about someone in our life. I wanted to just skip it but I’m glad I didn’t.

© 2024 Sexton JB et al. *JAMA Network Open.*

The exercises and feedback on research supporting integrating them into a routine

The exercises and tools were very good. Hearing the science behind the work was very compelling. Great presenter as well!

The exercises on gratitude and self-compassion were very therapeutic and something that I could see incorporating into my everyday life/ work

The fact that it made me feel better during each session. I enjoyed writing a letter to me from the person I most admire. It was both an eye opener and satisfying.

The fact that Y'all are making this content available is just awesome. I hope to use this content in my work!

The facts and how real it is.

The feedback

The first and second presentations were my favorite. I was grateful for the reminder to be thankful. I saw how taking just a few minutes in a day to do this could have long lasting and meaningful benefit.

The framework of how the sessions were set up were great. I enjoyed that each session we took we would receive feedback based on what we talked about.

The Gratitude exercise and awe--have discussed both of these with patients this past week.

The gratitude letter - I plan to continue writing a letter every four weeks. Most gratitude exercises encourage writing daily. I find this overwhelming and slightly irritating. Once a monthly seems doable and helpful.

The gratitude piece was terrifically useful. All of it was interesting and I will go back at watch again!

The gratitude section really spoke to me the most. I loved the video where they wrote letters to loved ones. I have shared this with my friends and family.

The gratitude tool was nice because it made me feel good and it made the recipient feel good. I loved all of the research that has gone into these tools and reframing burnout. I cannot wait to share with my team. Kudos!

The gratitude video was refreshing to watch!

The great examples.

the history of the scenarios and how they affect us all

The illustrations and practical applications

The impact I felt from the awe session.

The impact of changing the mindset to a positive perspective.

The impact of gratitude and ways to show gratitude

The importance of our personal well being on all aspects of our work and how simple changes can have a big effect.

The lead speaker was so engaging, listened and responded well, and gave context above and beyond what was on the slides (pet peeve = reading the slides)

The letter writing in the first session was very powerful. The continued reinforcement of self-praise and praise of others was very helpful

The link for seeing how stressed you are and what the level of work life is. I was able to share with my coworkers.

© 2024 Sexton JB et al. *JAMA Network Open.*

The literature reviews and the activities, the clinical practical application and some space to see the application to live at home

The many tips and suggestions that were provided t/o each session. I love seeing the research studies and the science behind why various strategies work. The slides were engaging and well put together.

The moderators were great and so knowledgeable. It has been so worth my time. I have a ways to get my work-life balance where it should be and this was a great start.

The most helpful during the series is how much the little tools really changed how I feel and have a different outlook at life.

The most helpful portion in this series was the self-compassion. I am very bad at that. I really like that you provided books and tools to help get better at it.

The most helpful thing from this series was learning to look at things from a different perspective, as well as some of the coping skills/tools provided.

The most helpful thing was turning off the chat feature so it didn't distract from the presentation of information. The most interesting was the passion and excitement felt by the presenters on their topics. Having the evidence to back up the tools will assist in implementation at my organization.

The most interesting aspect for myself was how new the application of these topics are.

The most surprising to me was the survey questions. I was shocked how impacted my mood and wellbeing actually was by my job. Made me pay extra attentions to tools and reflect on sessions, as I do not want to have my well being impacted to that extent.

The new view to look at work life balance.

the picture demonstrations were engaging, especially those used for the "Awe" segment

The pictures and the uplifting tones of the speakers

The power points and looking at the research

The presentation style- liked the bit size tasks, and the research to support it. A simple letter of gratitude and in the impact it can have

The presenters were awesome

The program started off strong and kept getting better! Wow!   The information was extremely helpful. Using information presented in the Work Life Integration, I queried my team and requested their feedback on three things that are going well and one thing that could be better. The team was asked to respond by Thursday of this week. Looking forward to the results!

The questionnaire

The RAFT format! I am both learning the tools and using them. I can't wait to bring these tools to my colleagues. Thank you all so much!

The realistic examples and goals for self-care and work-life balance. Gratitude was one of my favorite sessions. I feel I need to start a gratitude journal and remind myself of all the great things in my life and around me that I sometimes take for granted. I need to feel more pleasure and less stress.

The references with evidence from published articles/studies. Tangible ways to use the information

The reflections, writing the letter, making the phone call, etc.

© 2024 Sexton JB et al. *JAMA Network Open.*

The reminders about the importance of self-compassion and how we talk to ourselves were most helpful.

The research (for the why), then the bite size tools to apply!

The research and information that has come out of COVID. It's applicable to real life work in an academic medical center (anywhere really). It helps to reinforce the learnings from previous Duke/Bryan Sexton lessons.

The research and recordings

The research and science behind why we should be more thoughtful about caring for ourselves.

The research backed evidence behind the recommendations for well-being. Very impressive!

The research data

The research is incredibly inspiring and the group level and resources will help me tremendously as I work with others at each of my care sites. I plan to watch the sessions I've missed later this afternoon.

the research supporting the recommendations, and the short activities that can help

The research that shows the impact of these techniques

The research to support the work and the tools. Everything!

The research was compelling and I appreciated the self-assessments!

The science backing up the module content!

The science behind the information.

The self-compassion practices

The self-assessments

The self-compassion subject was an ah ha moment for me. I can be compassionate with others but I beat myself up for the same thing.

The self-compassion & awe and wonder presentations really struck me the most.

The self-compassion talk was the most useful for me. We are often way too hard on ourselves and need to extend grace to ourselves as much as we extend grace to others.

The self-compassion. I like the awe and wonder and feel I will use this as well as gratitude.

The self-compassion strategies shared were very valuable and I loved the section on awe &wonder. I can't wait to develop a lunch and learn for my organization highlighting it after learning about it and the research base behind it.

the series was very helpful and helped me identify MY effective coping skills for my home caregiving burnout issues

The session on gratitude was the most interesting and new to me.

The session on Awe was different from anything else I have experienced in this space. Thank you Made a huge impact, I spent the weekend reflecting remembering and finally ordered a print picture of one of the vistas for my husband’s birthday present. Burn out is hitting our marriage at the moment and using and applying these resources there to has helped me the last few days, I think we are OK

The session on awe was most interesting to me.

The sessions have been helpful, engaging, informative. I appreciate the journal resources.

© 2024 Sexton JB et al. *JAMA Network Open.*

The sessions on Gratitude, Self-Compassion, and Awe & Wonder. It was all helpful but those three helped me the most. I took these sessions during a really difficult and long (57 hrs.) week, actually second week in a row like that, and am surprised that I was able to get as much out of this as I was due to distractions of all I needed to get done for work. I really want to watch again and get my husband to watch with me. I suggested this to another leader in our dept also. Plan to use the three good things and one to do better with dept meetings.

The sessions that I got the most from were Work-Life balance and Self Compassion. I liked the offering of available resources that were given in the Self Compassion session.

The sessions were very beneficial to evaluate my life, personally and professionally, to see areas that need improvement. The webinars gave me insight and resources to help me. I have learned that weekends are for bike rides and walks. This helps me spend valuable time with my family. I am still working on how to incorporate some of this during the week.

The sessions were very helpful. I can't pinpoint a specific lesson. All sessions were insightful. Thanks again for this.

the short evidence-based research that displays LT benefits

The small tips and changes that can make a huge difference

The small workable tools one can use on a daily basis to avoid burn out. The tools did not take a large amount of time out of the day and can easily be implemented in a realistic working environment.

The small, short interventions provided that can have such a big impact on well-being.

The speaker's interaction and approach! They did wonderfully!

The speakers were wonderful and very informative. They really made the information interesting and I looked forward to the next section.

The statistics and the description of emotional exhaustion

The statistics were impressive. I have started journaling since the sessions as I feel this truly helps me.

The studies and examples of applications o

The studies presented, the awe and wonder scenes, tools.

The suggestion to write a gratitude letter (it was well-received by the person I wrote the letter to), the activity where a loved one practiced compassion with us but it was really self-compassion, and the different ways in which we can experience awe (doesn't have to be in nature).

The support I felt knowing that I am allowed and even encouraged to scheduling “me” time

The things you can do each day to improve outlook

The time and reminder to take a pause, how powerful reflection time can help your well being.

The time checked out of work working on self

the time given during each session for the activity

The tools and activities. Research is good too when developing education for clinicians and leadership.

The tools provided to help implement the practices into everyday life

The tools that could be applied to all hours of the day, not just during work.

The tools to help cope and change the culture at work.

© 2024 Sexton JB et al. *JAMA Network Open.*

the tools to improve work life balance and to improve overall wellbeing

The tools used

The tools used.

The tools we used every day were most helpful for me to get into a healthier headspace, which allowed me to make some small, healthy changes to my weekday routine that improved my overall well-being.

The usable content in each presentation, I walked away from each session with specific actions to take to improve my well-being and the well-being of my colleagues.

The use of Awe.   Have not tried before.

The use of data was nice.

The videos and the speaker.

The wellbeing activity during each session brought a bit of calm to chaotic days.

The whole program is amazing. I particularly enjoy the data on how burnout, emotional exhaustion, etc. are clustered in work environments. These programs are great for helping people (I've gone through the course before) learn how to cultivate their positive emotions.

There were many ideas and supporting documents for the information that was presented. For example, not doing a Gratitude Journal (which I have tried to do in the past and fail by day 3) instead writing a letter. Huge satisfier for me.

There were many interesting things I learned. I liked the fact that changing the focus on leader rounding improved safety.

They provided short activities to complete that were proven to have lasting effects

thinking about the impact of awe and gratitude

Thinking of someone that has influenced me the stories are powerful and I enjoy watching them but sometimes struggle with actually seeing people in my organization have those conversations.

This could not have happened at a more appropriate time for me. Really going through some tough life changing events at work. Now I have a much better understanding of where I was at, how it happened and now what I can do about it. Thank you so much!!!

This course has been absolutely amazing and I hope that within a month+ I feel even better.

This has been amazing! I'm very interested to continue receiving this content!

This is a very good program

This is a very small piece of the sessions but one thing that stuck with me was from the work-life balance session when John used the example of feeling disassociated and thinking of people as just 'objects in the way'. Unfortunately, this resonated with how I've been feeling working in healthcare, despite doing indirect patient care.

This is the second session of this cohort, but the questions assume completion.

This series was very helpful, as it validated how I've been feeling at work as legitimate and bot just me being too sensitive or overthinking it.

This was a great program!

This was a great series!

© 2024 Sexton JB et al. *JAMA Network Open.*

this was a great session

This was a wonderful refresher after the resilience ambassador multi-day course before the pandemic

This was amazing to attend and experience your work and as a researcher I admire the line of clinically relevant research. Keep it going I can't wait to watch the last two recordings. I was away on business and was unable to attend live. Thanks for the opportunity to see the recordings

This was an amazing series that I am thrilled I stumbled upon during my research to support my colleagues.

This was an amazing series, which I did in my own time over the past few weeks, but enjoyed immensely. I love the bite size tools. I've had some trouble remembering each of them. Hoping getting all the resources on one page that I can bookmark will help. Make an App! I would dig it.

This was awesome 👏

This was excellent! Thank you for offering a life jacket. Work talks about this and schedules more meetings, and more courses to take with a high cost like $700/course- which is not affordable to all.

This was great; and I look forward to using the tools learned!

This was great. My emotional exhaustion decreased significantly. I have shared the links with several others. Thank you sharing this knowledge and for making the links available!

This was so helpful over all. I've already started making some of the changes

This was so impactful. After the first session I shared the link with 10 leaders that were unable to fit the live times in their day. I have continued to share and love the positive feedback. I also rewatched them at night with my husband and shared with my daughters that are in health care. I was surprised to see my sleep time improved in just one week. I have found myself singing. Pre pandemic I read a book every 1-2 days. I have not been able to read a book in over 2 years. My goal is to be able to focus and bring back one of my greatest joys. I went book shopping this weekend and renewed my library card today. I will also use what I learned and share with coworkers.

Thoroughly enjoyed this series

Thoroughly loved the peer reviewed research and the use of multimedia

Thought this was a very good seminar. I really appreciated having recordings after since I wasn’t able to get to the live sessions due to my work schedule.

Tips

Tips on bringing these tools back to the practice and helping others

To know I’m not alone in balancing work and life. Hearing ideas and strategies to provide positivity so my focus can adjust on behavior.   Still may not have work life balance, but not dragging every day now.

To know I’m not alone in my feeling of burnout and that I now have concrete ways I can consciously work on regaining the joy and purpose I once felt about my career as a social worker.

To know we are all going through this

To learn that my mindset can affect my attitude more than an actual situation.

to make a gratitude list

To realize, I'm not alone with my feelings. I love the strategies that can be done by anyone. I liked hearing the evidence behind the strategies as well. I was in awe of the winter's beauty this year, and now I know the benefit of doing an "awe walk." Thanks so much for the great presentations and content. Great presenting styles too.

© 2024 Sexton JB et al. *JAMA Network Open.*

Tools Repeat review of wellbeing questions.

Tools

Tools and resources. Performing activities helps to engrain in my head.

Tools and tips

Tools available to use for each category.

Tools to help myself balance and find peace

Truly I found it all extremely interesting & helpful- the easy concepts, the short lessons & activities. I wish our hospital would make the monthlies available for us.

Truly wonderful! Thanks so very much!!

trying to find ways to change work-life balance and still working on this

Trying to remember to find awe in the little things like when my child smiles or looking at nature while on a walk. Sometimes it’s hard to do that.

Understanding Awe. Have experienced the impact but framing and understanding its effectiveness. Also loved the exercise of what you would say to encourage a friend to find your positive self-talk or self-compassion. Great series!

valid research, interesting presentations, videos, and activities

Validation of burnout with others and science behind why.

Very engaging and interactive. Learned lots of things that can help on any day. Thank you!

Very enjoyable. Recommend for all.

Very helpful and a nice break from work

Very helpful and insightful

Very helpful in small sessions like this with great evidence and resources

Very helpful information. I was looking for something to share with new nurse graduates as they start their nursing careers. I currently have them complete the Science of Well-Being course offered by Yale on Coursera. This is much more manageable for their time demands.

Very helpful material. Thanks.

very helpful resources. wish I did this earlier! quick and easy ways to incorporate wellness daily

Very insightful!

Very inspirational

Very much enjoyed these.

Very well done and appreciate all the resources and tools provided.

Very well-done series.

Very well done! Very engaging and enjoyable!

© 2024 Sexton JB et al. *JAMA Network Open.*

Virtual classes since I would not have been able to attend otherwise. I enjoyed the format, group exercise, and allowing time for questions. The benefit of sleep really was a strong takeaway since I do wake up tired and drag before the work day starts. Been working on getting to bed early and clearing my, prepping after dinner for next morning so not as rushed. I also enjoyed the exhaustion tool and shared with a coworker in a similar position to keep her aware of factors we can influence/control.

WAYS TO BE MORE GRATEFUL

WAYS TO BE MORE GRATEFUL

Ways to destress

ways to utilize with my team. Bite-size nuggets to share on a weekly basis and reinforce during rounding.

We are working at our hospital to include regular group processing for proactive well-being. I am hoping I can find a way to integrate the lessons from this into our process. Thank you for all your hard work!

Well done. Need this weekly and ongoing instead of just 4 days. Feel like I need this every week for a few months to get back on track.

Wellness tips

What do you do when your leader(s) do not buy into any of this?

While it was encouraging to me to know that my emotional recovery score and my emotional thriving scores were very high, it was no surprise to me that my work/life balance score was also very high. I'm glad to know that I can at least recover and continue to thrive, but I do realize that I need to better balance my work life and my personal life for my own emotional well-being. The ideas to do this that were presented were very helpful, and I'm now making a more conscious effort to use them so that I can be better for others. I've actually even left things until the next day recently so that I'm not sacrificing my personal time with myself and loved ones. Yay me!

Will recommend to others.

Wish I could do the monthly.

WL balance talk

Wonderful and thoughtful Information

Wonderful nuggets, easily accessible

wonderful sessions!

work-life balance

Work-life balance

Work-life balance

Work-life balance and definition of burnout also being kind to yourself

work-life balance video

Work-life balance.

work-life balance

Would have benefited from going in a little more on the specific studies. Commenting on if there was a placebo effect seen and things like that

© 2024 Sexton JB et al. *JAMA Network Open.*

You rock in all of it!! So relevant, timely, and supportive!

You two slate an awesome duo! Thanks so much for putting on such a great series. I'll miss it!

Your topics finally are understood. I have been working on gratitude and other measures during COVID as an Infection Preventionist. It feels as if the science presented helped me to understand what changes have accrued in me. I feel the tools and resources will positively impact me to make a positive change back to myself as I was prior to Covid and a life-changing event that occurred as the pandemic hit.

© 2024 Sexton JB et al. *JAMA Network Open.*
