# Supplementary material for: Well-Being Outcomes of Health Care Workers After a 5-Hour Continuing Education Intervention: The WELL-B Randomized Clinical Trial
Source: JAMA Netw Open. 2024 Sep 19;7(9):e2434362. doi: 10.1001/jamanetworkopen.2024.34362 (PMC11413716; doi:10.1001/jamanetworkopen.2024.34362)
Supplement: Supplement 3. — Data Sharing Statement [file jamanetwopen-e2434362-s003.pdf]

Data Sharing Statement

Sexton. Well-Being Outcomes of Health Care Workers After a 5-Hour Continuing Education Intervention. *JAMA Netw Open*. Published September 19, 2024.
doi:10.1001/jamanetworkopen.2024.34362

Data
Data available: Yes
Data types: Deidentified participant data
How to access data: reasonable requests can be made to bryan.sexton@duke.edu, the corresponding author.
When available: With publication

Supporting Documents
Document types: Informed consent form
How to access documents: reasonable requests can be made to bryan.sexton@duke.edu, the corresponding author.
When available: With publication

Additional Information
Who can access the data: researchers whose proposed use of the data has been approved
Types of analyses: for a specified purpose
Mechanisms of data availability: with a signed data access agreement
